# Supplementary material for: An injury-associated lobular microniche is associated with the classical tumor cell phenotype in pancreatic cancer
Source: Nat Commun. 2025 Sep 26;16:8307. doi: 10.1038/s41467-025-63864-7 (PMC12475445; doi:10.1038/s41467-025-63864-7)
Supplement: Supplementary file 1 — Supplementary Information [file 41467_2025_63864_MOESM1_ESM.pdf]

An injury-associated lobular microniche is associated with the classical tumor cell phenotype in pancreatic cancer

Sara Söderqvist[1], Annika Viljamaa[1], Natalie Geyer[1], Anna-Lena Keller[1], Kseniya Ruksha[1], Carina Strell[2,3], Neda Hekmati[3], Alexandra Niculae[1], Jennie Engstrand[1], Ernesto Sparrelid[1], Caroline Salmén[1], Tânia D.F. Costa[4], Miao Zhao[3], Staffan Strömblad[4], Argyro Zacharouli[5], Poya Ghorbani[1], Sara Harrizi[1], Yousra Hamidi[1], Olga Khorosjutina[6], Stefina Milanova[6], Bernhard Schmierer[6], Béla Bozóky[5], Carlos Fernández Moro[1,5,7#], Marco Gerling[1,8,#,*]

[#] these authors jointly supervised the work

1. Department of Clinical Science, Intervention and Technology – CLINTEC, Karolinska Institutet, 14183 Huddinge, Sweden
2. Centre for Cancer Biomarkers - CCBIO, Department of Clinical Medicine, University of Bergen, 5020 Bergen, Norway
3. Department of Immunology, Genetics and Pathology, Uppsala University, 75185 Uppsala, Sweden
4. Department of Medicine, Huddinge - MedH, Karolinska Institutet, 14183 Huddinge, Sweden
5. Department of Clinical Pathology and Cancer Diagnostics, Karolinska University Hospital, Stockholm, 14186, Sweden
6. CRISPR Functional Genomics, SciLifeLab and Department of Medical Biochemistry and Biophysics, Karolinska Institutet, 17165 Solna, Sweden
7. Department of Laboratory Medicine, Division of Pathology, Karolinska Institutet, 14186, Stockholm, Sweden
8. Theme Cancer, Karolinska University Hospital, 17 176 Solna, Sweden

[*]To whom correspondence should be addressed:
Dr. med. Marco Gerling
marco.gerling@ki.se
Karolinska Institutet
Department of Clinical Science, Intervention and Technology – CLINTEC
NEO
Hälsovägen 7
141 83 Huddinge, Sweden

Supplementary Information:
Supplementary Figures 1-20
Supplementary Tables 1-8

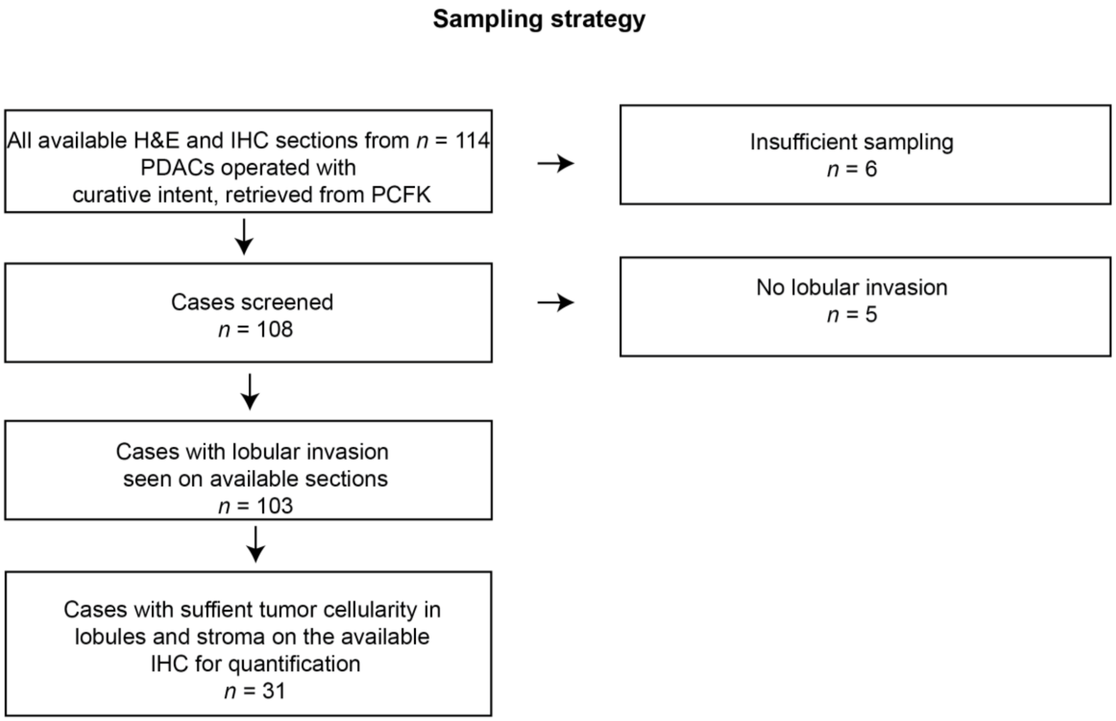

Supplementary Figure 1: Schematic of the sampling processing, related to Fig. 1d. *N* = 114 pancreatic ductal adenocarcinomas (PDACs) were identified (operated in the years 2017 and 2020 at Karolinska University Hospital, Stockholm, Sweden), from which all Hematoxylin and eosin (H&E) and immunohistochemistry (IHC) sections were retrieved. The years for inclusion were randomly chosen, and cases were consecutive. Six cases were insufficiently sampled to determine the presence of lobular invasion, leaving *n* = 108 cases for screening of lobular invasion. Five cases showed no signs of lobular invasion in the available sections, while *n* = 103 cases had evident tumor cells within lobules. Insufficient sampling was due to fewer than ten available sections being retrieved, or only non-parenchymal tissue structures, such as lymph nodes, adipose tissue, and duodenum, being captured on the available slides. Cases with no apparent lobular invasion entailed, for example, cases with completely stroma-transformed regions where tumor cells were scarce, and tumors with extensive pools of mucin. *N* = 31 cases with abundant tumor cellularity were characterized in depth for the expression of subtype-related IHC markers. PCFK: Pathology Core Facility Karolinska.

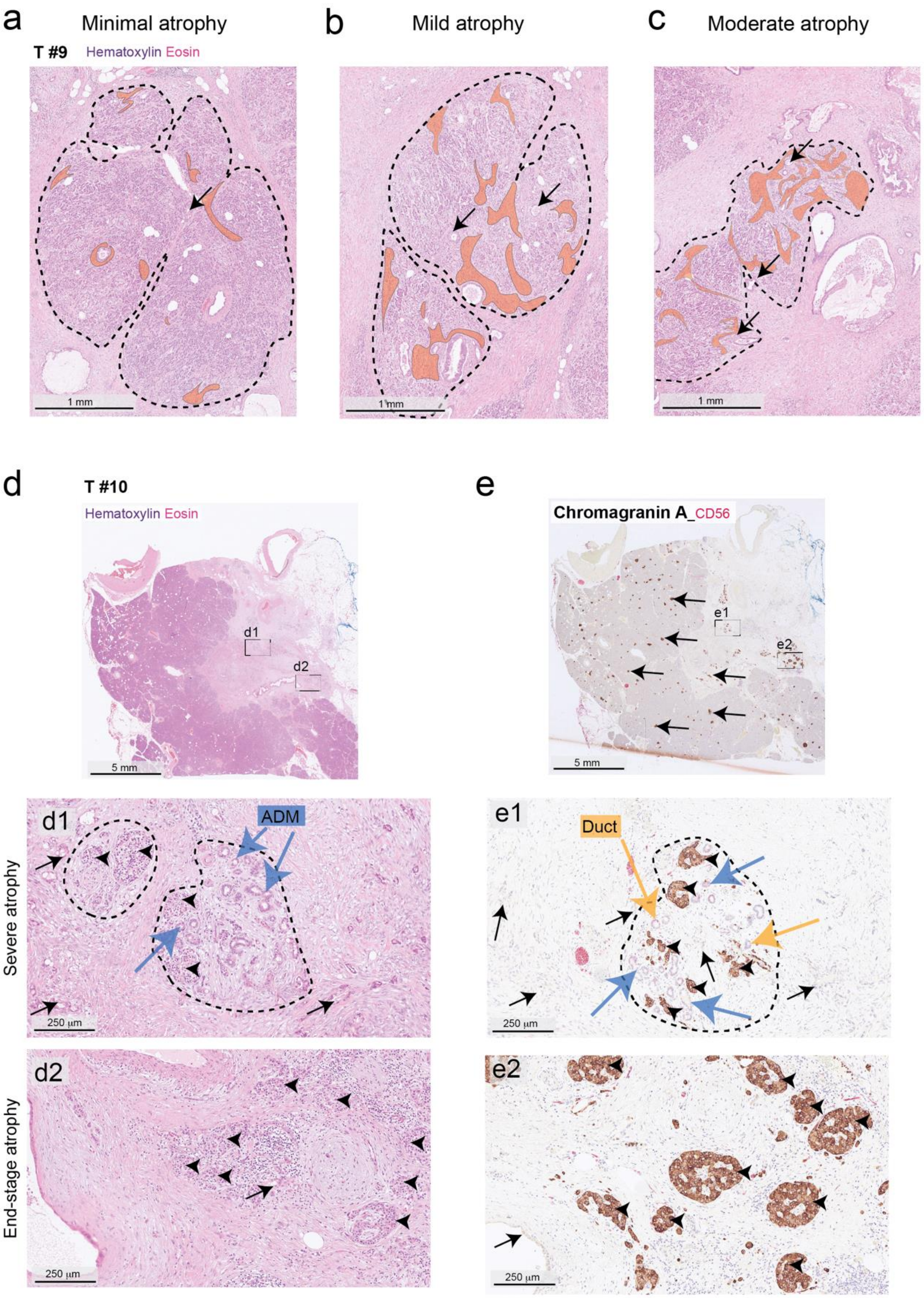

Supplementary Figure 2: Atrophy of the pancreatic parenchyma and tumor progression. a)-c) Representative hematoxylin and eosin (H&E) stain of increasing lobular atrophy, accompanied by stromal expansion (light orange overlay) and regions of tumor invasion (black arrows). (**a**) Lobule with minimal atrophy. As the degree of lobular atrophy increases to mild (**b**) and moderate **(c)**, the parenchyma shrinks with more prominent stroma, which eventually results in near-complete stromal transformation in end-stage atrophy. **d)** H&E and **e)** chromogranin A

stain (brown, endocrine cells) showing severe (**d1** and **e1**) or end-stage (**d2** and **e2**) lobular atrophy. Blue arrows: Acinar-to-ductal metaplasia (ADM), yellow arrows: ductular remnants, black arrowheads: endocrine islets of Langerhans, black arrows: tumor. Dashed line: pancreatic lobules. Additional clinical stain not considered further here: cluster of differentiation 56 (CD56). **a)-e)**: Representative of n = 31 tumors.

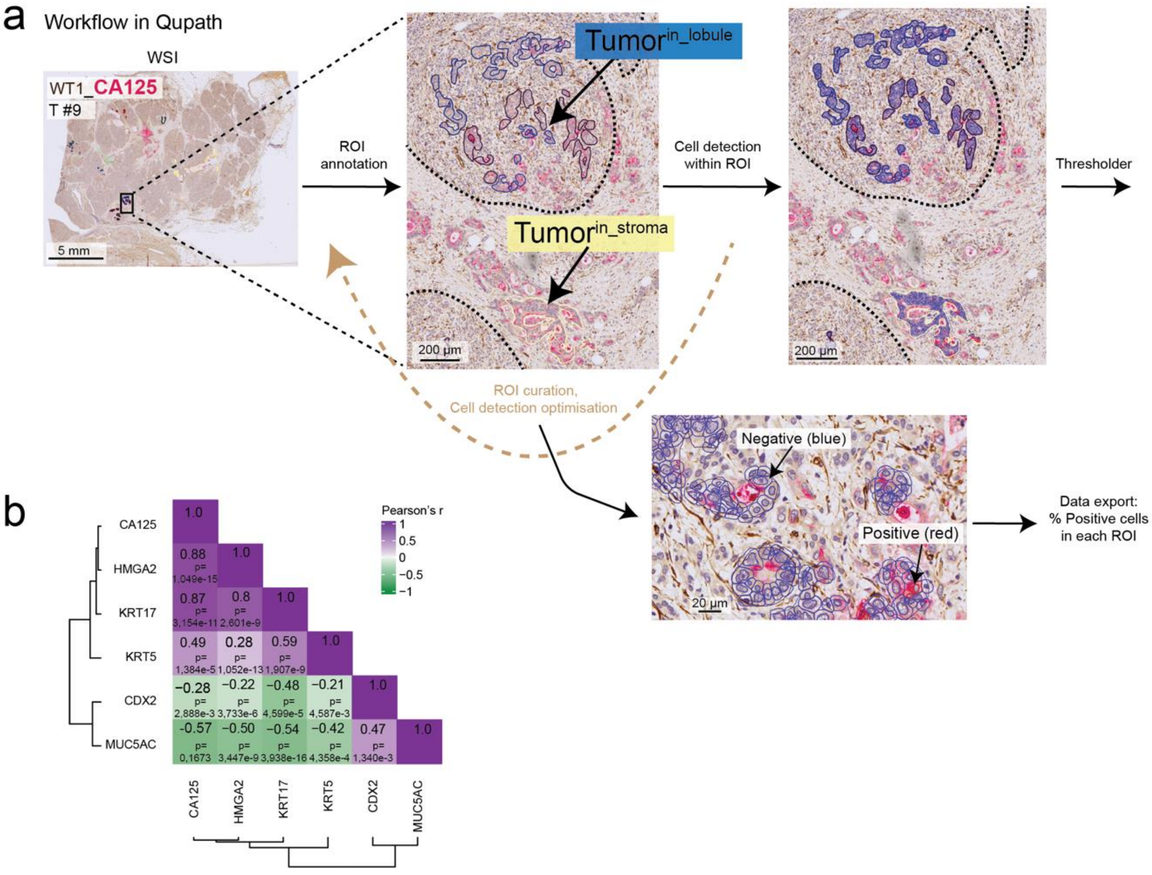

Supplementary Figure 3: QuPath annotation workflow for analyzing compartment-dependent heterogeneity and marker correlation. Related to Figure 2. **a)** Overview of the manual annotation workflow in QuPath, here exemplified by a whole-slide image (WSI) of an immunohistochemistry (IHC) stain for carbohydrate antigen (CA)125 (red, cytoplasmic) co-stained with a stromal marker, WT1 transcription factor (WT1, brown). Up to n = 18 regions of interest (ROIs) were blindly selected on a matched hematoxylin and eosin (H&E) stain and subsequently annotated for each IHC WSI. ROIs were dichotomized to tumor^{in_lobules} and tumor^{in_stroma}. **b)** Correlogram of all markers quantified, based on all quantified ROIs (n = 2105 ROIs). Classical markers: caudal type homeobox 2 (CDX2) and mucin 5AC oligomeric mucus/gel forming (MUC5AC). Basal markers: Keratin (KRT)17, KRT5, high mobility group AT-hook 2 (HMGA2), and carbohydrate antigen (CA)125. Each cell of the correlogram contain the Pearson's correlation coefficient (r) on top and the p-value tested with 95% confidence interval level based on the Pearsons's correlation on the bottom. Data from n = 31 tumors. Source data are provided as a Source Data file.

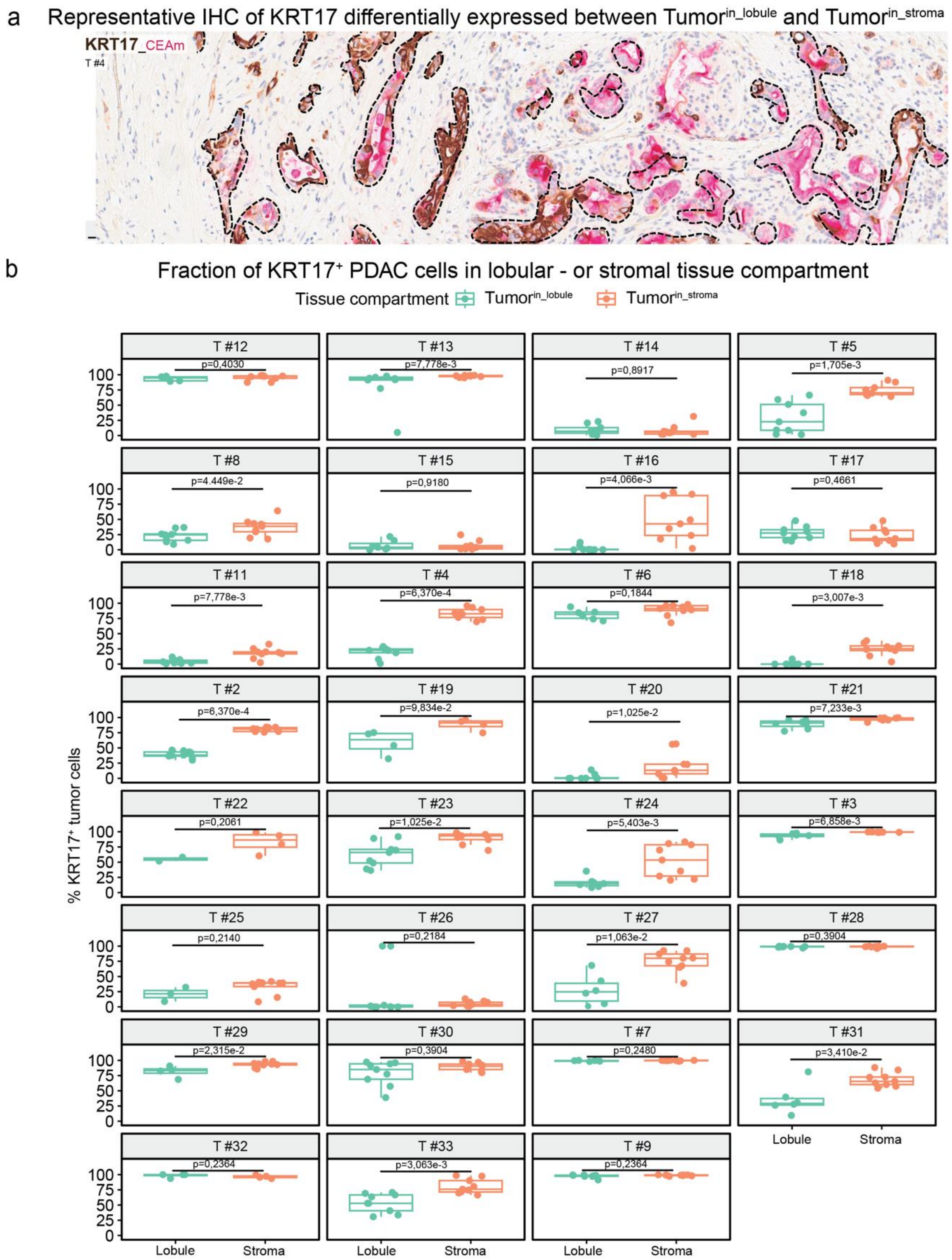

Supplementary Figure 4: Keratin 17 is more abundantly expressed by tumor cells in the stroma than in the pancreatic lobules. a) Representative immunohistochemistry (IHC) of the basal marker keratin (KRT)17 (brown, cytoplasmic) co-stained with monoclonal carcinoembryonic antigen (CEAm, red, cytoplasmic) differentially expressed between lobular and stromal invasion. Stroma toward left, pancreatic lobule toward right. Black dashed lines: tumor. Representative for *n* = 31 tumors. **b)** Unpaired two-tailed Wilcoxon rank sum test with Benjamini-

Hochberg (BH) correction for multiple testing of quantifications for each tumor (T #) immunolabelled with KRT17_CEAm. Up to nine regions of interest (ROIs) per compartment were manually annotated in QuPath to include tumor cells in the respective compartment, and subsequently thresholded for the brown channel to stratify KRT17-positive and KRT17-negative tumor cell detections. Each dot represents the fraction of $KRT17^+$ tumor cells in one individual ROI. Box-and-Whisker plots show the median (line), the interquartile range (IQR, box), minimum and maximum values within 1.5 times IQR from the first and third quartile (whiskers), and individual data points. BH-corrected p-values are stated. Source data are provided as a Source Data file.

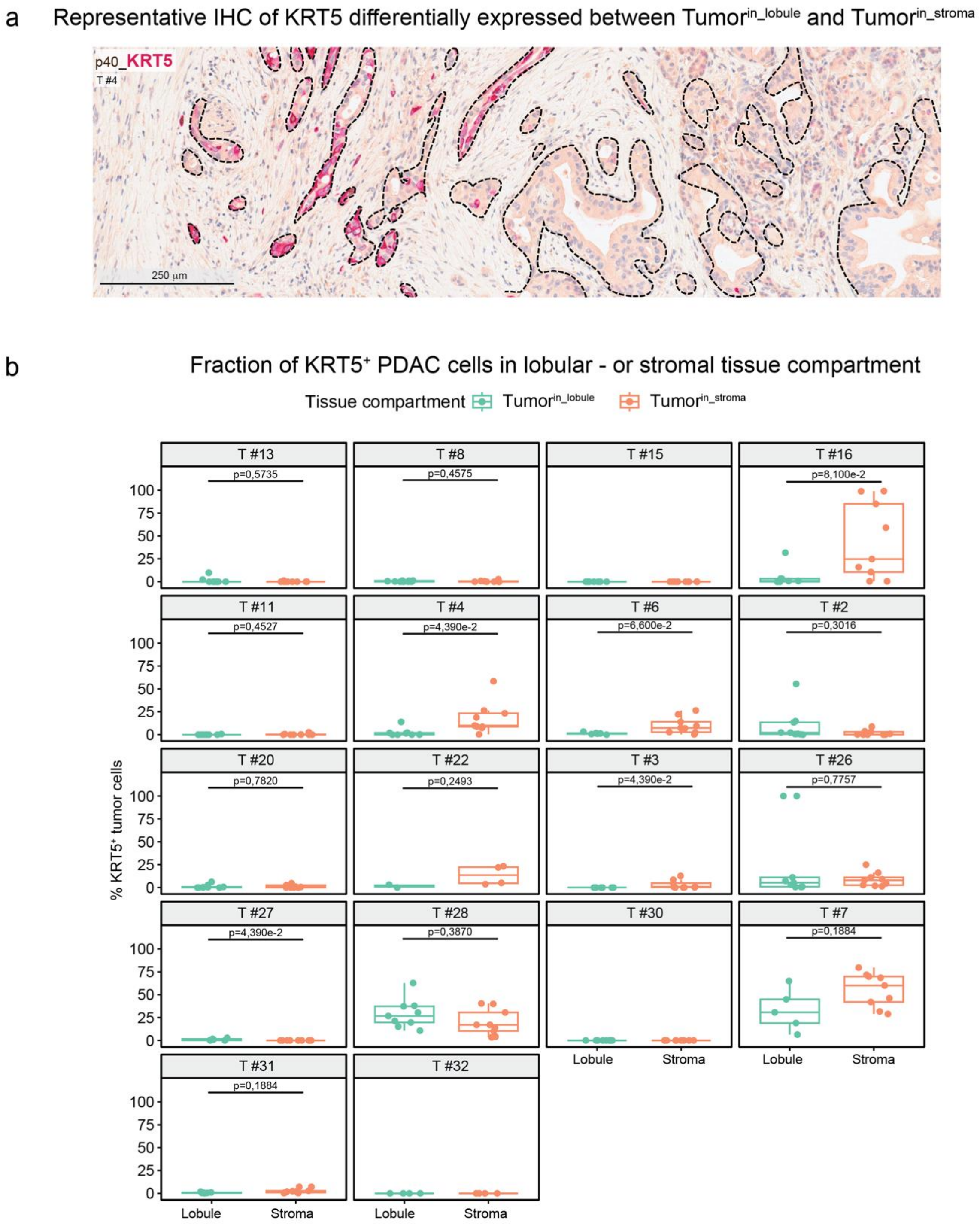

Supplementary Figure 5: Keratin 5 is more abundantly expressed by tumor cells in stroma than in pancreatic lobules. a) Representative immunohistochemistry (IHC) of the basal marker keratin (KRT)5 (red, cytoplasmic) co-stained with p40 (brown, nuclear) differentially expressed between lobular and stromal invasion. Stroma toward left and pancreatic lobule toward right. Black dashed lines: tumor. Representative for n = 18 tumors. **b)** Unpaired two-tailed Wilcoxon rank sum test, with Benjamini-Hochberg (BH) correction for multiple testing of quantifications for each tumor (T #) clinically immunolabelled with p40_KRT5. Up to nine regions of interest (ROIs) per compartment were manually annotated in QuPath to include tumor cells in the respective compartment, and subsequently thresholded for the red channel to stratify KRT5-positive and negative tumor cell detections. Each dot represents the fraction of KRT5^{+} tumor cells in one individual ROI. Box-and-Whisker plots show the median (line), the

interquartile range (IQR, box), minimum and maximum values within 1.5 times IQR from the first and third quartile (whiskers), and individual data points. BH-corrected p-values are stated. Source data are provided as a Source Data file.

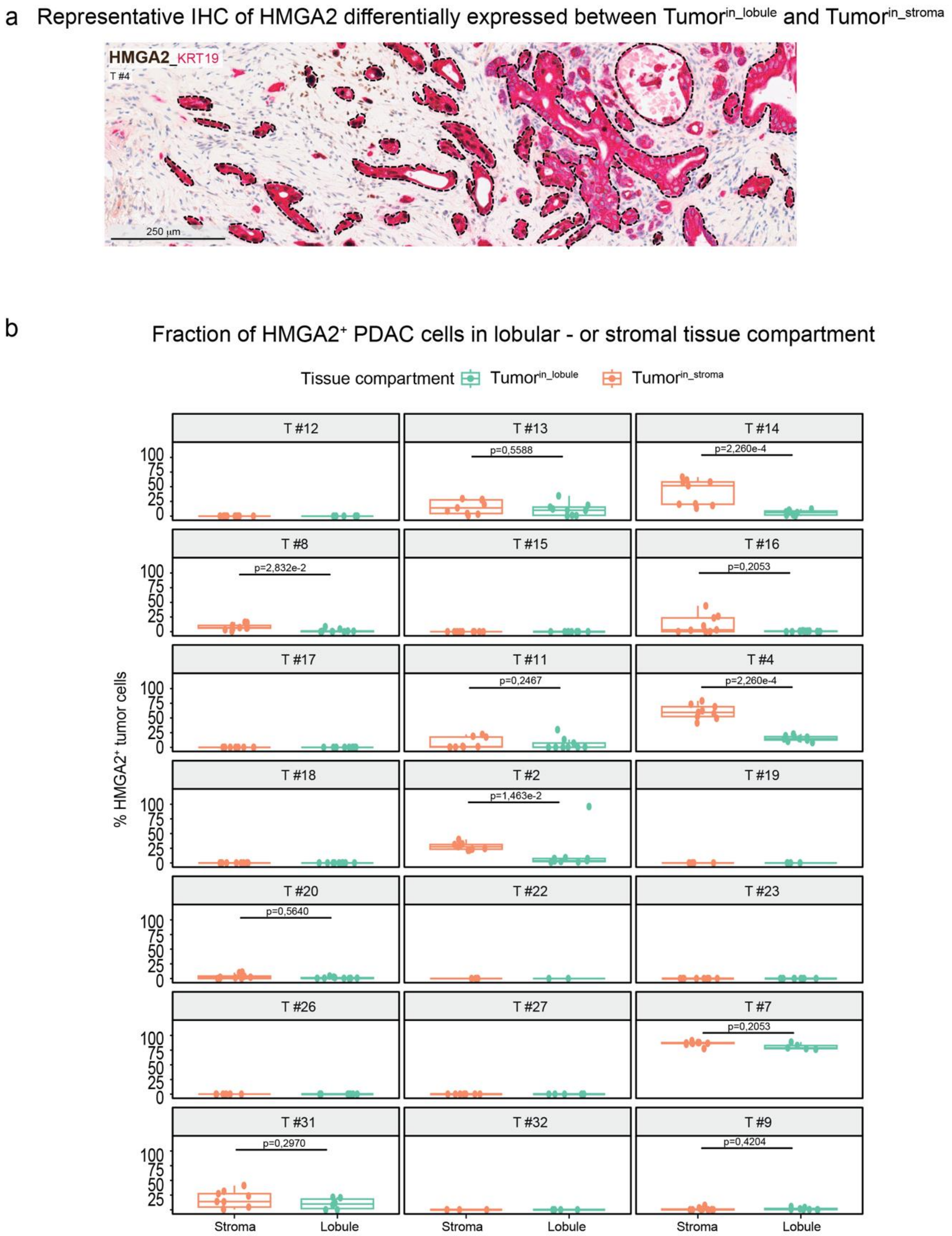

Supplementary Figure 6: High-mobility group AT-hook 2 is more abundantly expressed by tumor cells in stroma than in pancreatic lobules. a) Representative immunohistochemistry (IHC) of the basal marker high mobility group AT-hook 2 (HMGA2, brown, nuclear) co-stained with Keratin (KRT)19 (red, cytoplasmic) differentially expressed between lobular and stromal invasion. Stroma toward left and pancreatic lobule toward right. Black dashed lines: tumor. Representative for n = 21 tumors. **b)** Unpaired two-tailed Wilcoxon rank sum test, with Benjamini-Hochberg (BH) correction for multiple testing of quantifications for each tumor (T #) clinically immunolabelled with HMGA2_KRT19. Up to nine regions of interest (ROIs) per compartment were manually

annotated in QuPath to include tumor cells in the respective compartment, and subsequently thresholded for the brown channel to stratify HMGA2 positive and negative tumor cell detections. Each dot represents the fraction of $HMGA2^+$ tumor cells in one individual ROI. Box-and-Whisker plots show the median (line), the interquartile range (IQR, box), minimum and maximum values within 1.5 times IQR from the first and third quartile (whiskers), and individual data points. BH-corrected p-values are stated. Source data are provided as a Source Data file.

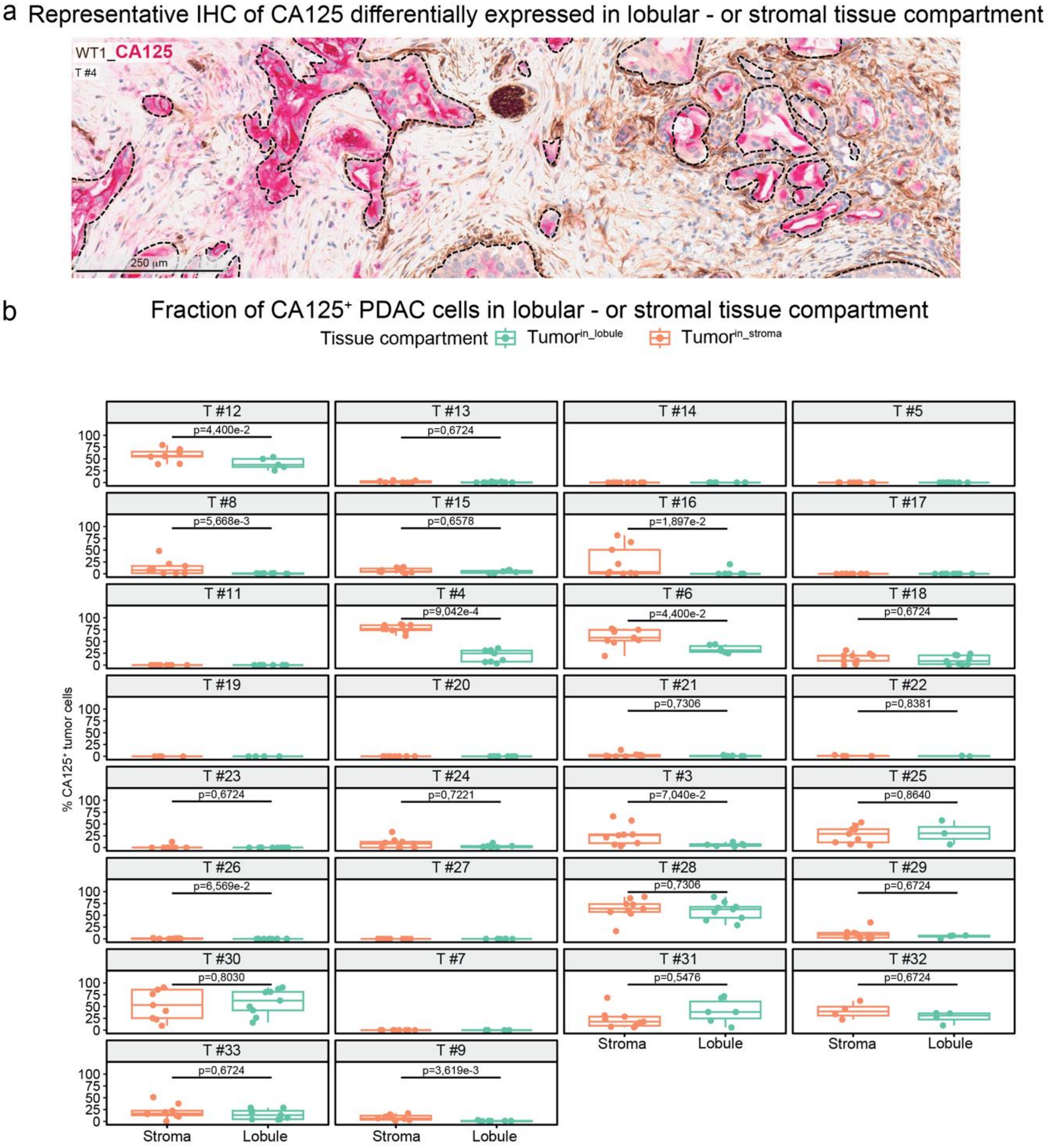

Supplementary Figure 7: Mucin 16/carbohydrate antigen 125 is more abundantly expressed by tumor cells in stroma than in pancreatic lobules. a) Representative immunohistochemistry (IHC) of the basal marker carbohydrate antigen (CA)125 (red, cytoplasmic) co-stained with WT1 transcription factor (WT1, brown, cytoplasmic) differentially expressed between lobular and stromal invasion. Stroma toward left and pancreatic lobule toward right. Black dashed lines: tumor. Representative for *n* = 30 tumors. **b)** Unpaired two-tailed Wilcoxon rank sum test, with Benjamini-Hochberg (BH) correction for multiple testing of quantifications for each tumor (T #) clinically immunolabelled with WT1_CA125. Up to nine regions of interest (ROIs) per compartment were manually annotated in QuPath to include tumor cells in the respective compartment, and subsequently thresholded for the red channel to stratify CA125 positive and negative tumor cell detections. Each dot represents the fraction of $CA125^+$ tumor cells in one individual ROI. Box-and-Whisker plots show the median (line), the interquartile range (IQR, box), minimum and maximum values within 1.5 times IQR from the first and third quartile (whiskers), and individual data points. BH-corrected p-values are stated. Source data are provided as a Source Data file.

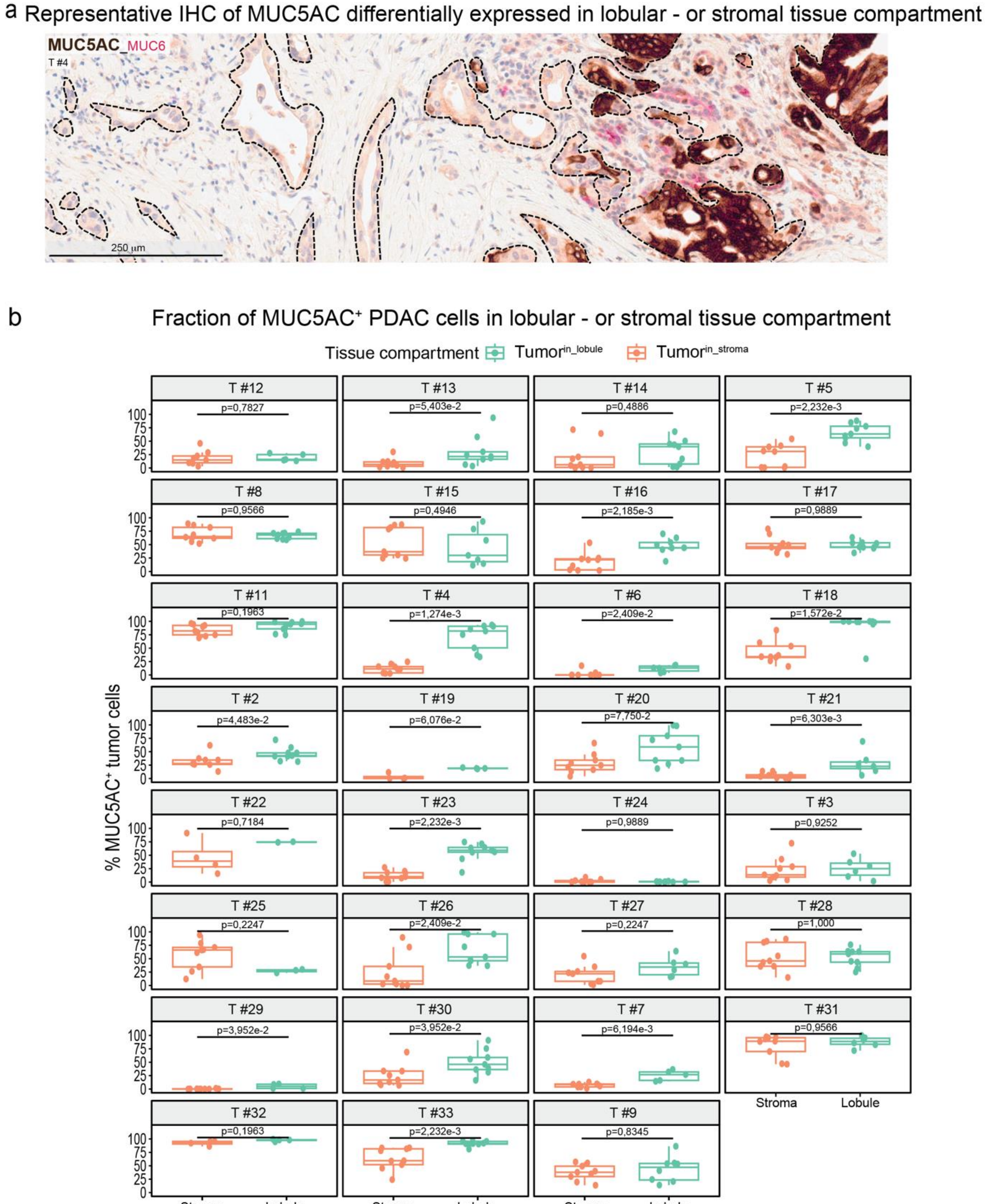

Supplementary Figure 8: Mucin 5AC is more abundantly expressed by tumor cells in pancreatic lobules than in stroma. a) Representative immunohistochemistry (IHC) of the classical marker mucin (MUC) 5AC oligomeric mucus/gel forming (MUC5AC, brown, cytoplasmic) co-stained with MUC6 (red, cytoplasmic) differentially expressed between lobular and stromal invasion. Stroma toward left and pancreatic lobule toward right. Black dashed lines: tumor. Representative for n = 31 tumors. **b)** Unpaired two-tailed Wilcoxon rank sum test, with Benjamini-Hochberg (BH) correction for multiple testing of quantifications for each tumor (T #) clinically

immunolabelled with MUC5AC_MUC6. Up to nine regions of interest (ROIs) per compartment were manually annotated in QuPath to include tumor cells in the respective compartment, and subsequently thresholded for the brown channel to stratify MUC5AC positive and negative tumor cell detections. Each dot represents the fraction of MUC5AC$^+$ tumor cells in one individual ROI. Box-and-Whisker plots show the median (line), the interquartile range (IQR, box), minimum and maximum values within 1.5 times IQR from the first and third quartile (whiskers), and individual data points. BH-corrected p-values are stated. Source data are provided as a Source Data file.

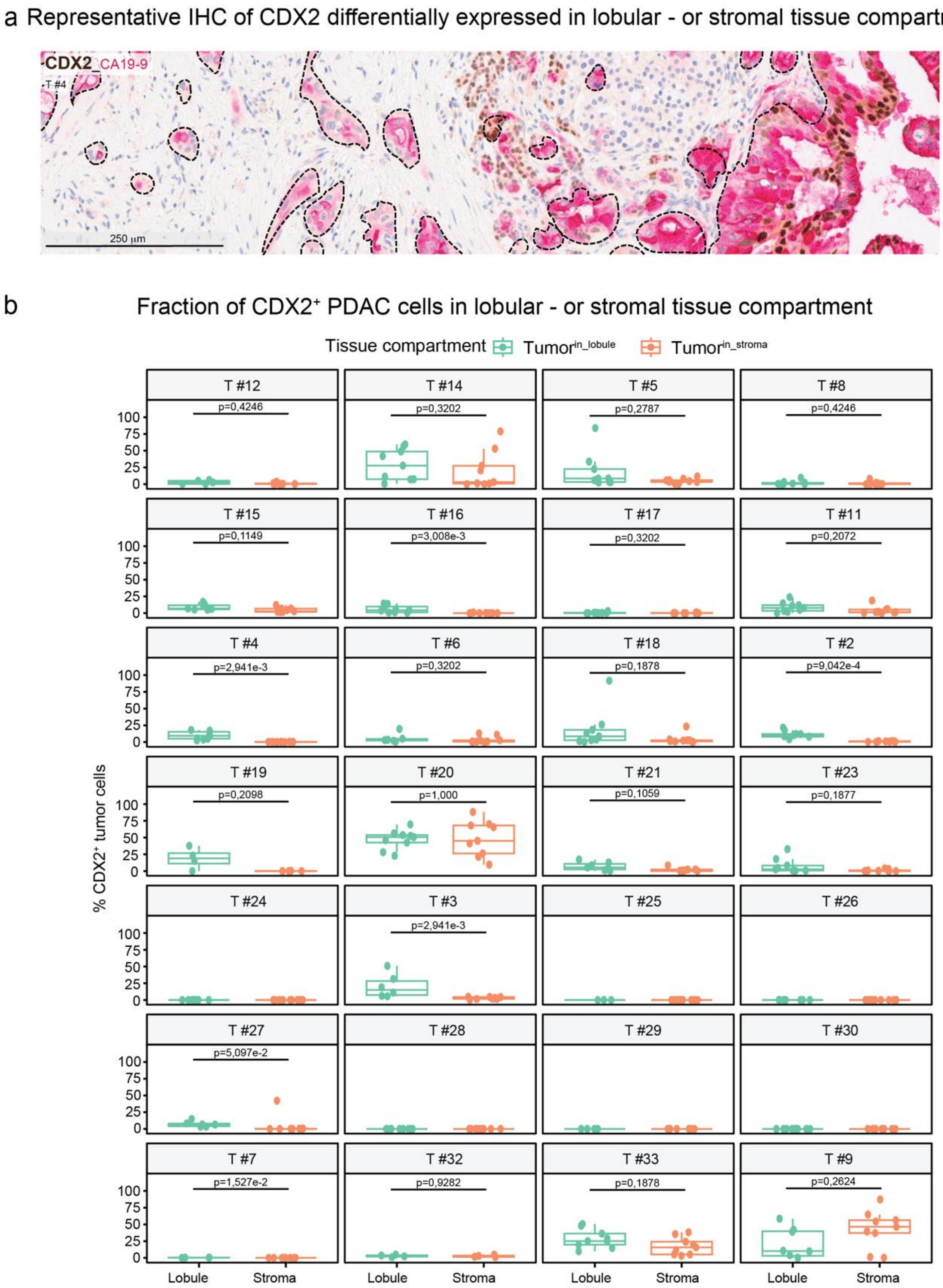

Supplementary Figure 9: Caudal type homeobox 2 is more abundantly expressed by tumor cells in pancreatic lobules than in stroma. a) Representative immunohistochemistry (IHC) of the classical marker caudal type homeobox 2 (CDX2; brown, nuclear) co-stained with carbohydrate antigen (CA)19-9 (red, cytoplasmic) differentially expressed between lobular and stromal invasion. Stroma toward left and pancreatic lobule toward right. Black dashed lines: tumor. Representative for *n* = 28 tumors. **b)** Unpaired two-tailed Wilcoxon rank sum test, with Benjamini-Hochberg (BH) correction for multiple testing of quantifications for each tumor (T #) clinically immunolabelled with CDX2_CA19-9. Up to nine regions of interest (ROIs) per compartment were manually

annotated in QuPath to include tumor cells in the respective compartment, and subsequently thresholded for the brown channel to stratify CDX2 positive and negative tumor cell detections. Each dot represents the fraction of CDX2^{+} tumor cells in one individual ROI. Box-and-Whisker plots show the median (line), the interquartile range (IQR, box), minimum and maximum values within 1.5 times IQR from the first and third quartile (whiskers), and individual data points. BH-corrected p-values are stated. Source data are provided as a Source Data file.

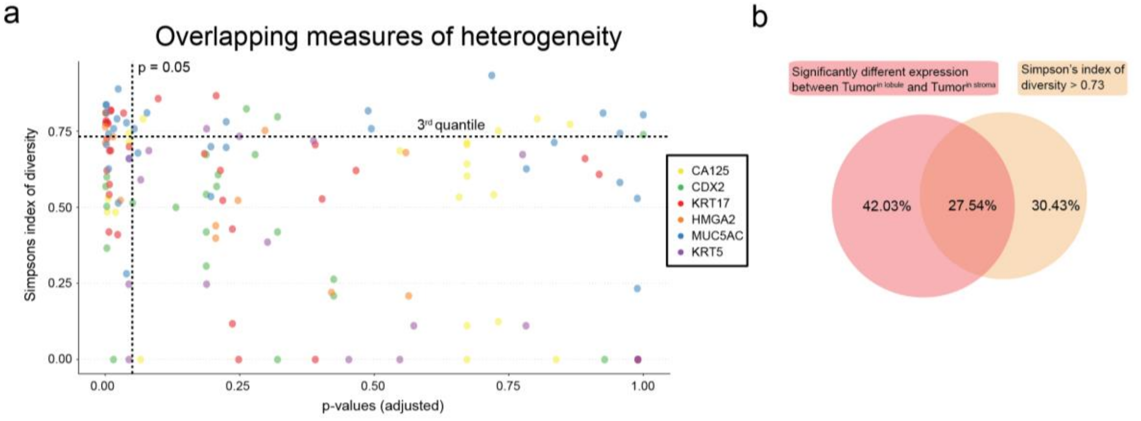

Supplementary Figure 10: Lobular vs. stromal tumor location explains some but not all phenotypic heterogeneity. a) Benjamini-Hochberg adjusted p-values from Wilcoxon tests **(Supplementary Fig. 4-9**) against Simpson's index of diversity. The Simpson index of diversity represents the probability of retrieving two random regions of interest from each stain and tumor in different abundance categories, irrespective of their tissue compartment. Dots within the upper left square represent cases and stains with significantly different expression between lobular and stromal compartments and with high overall heterogeneity according to Simpson's index. CA125: Carbohydrate antigen 125, CDX2: Caudal type homeobox 2, KRT: Keratin, HMGA2: High mobility group AT-hook 2, MUC5AC: Mucin 5AC oligomeric mucus/gel forming. **b)** Proportional Venn diagram of the same data as in (a) showing cases and stains that have both high spatially dependent and overall heterogeneity. **a)**, **b)**: Data from n = 31 tumors. Source data are provided as a Source Data file.

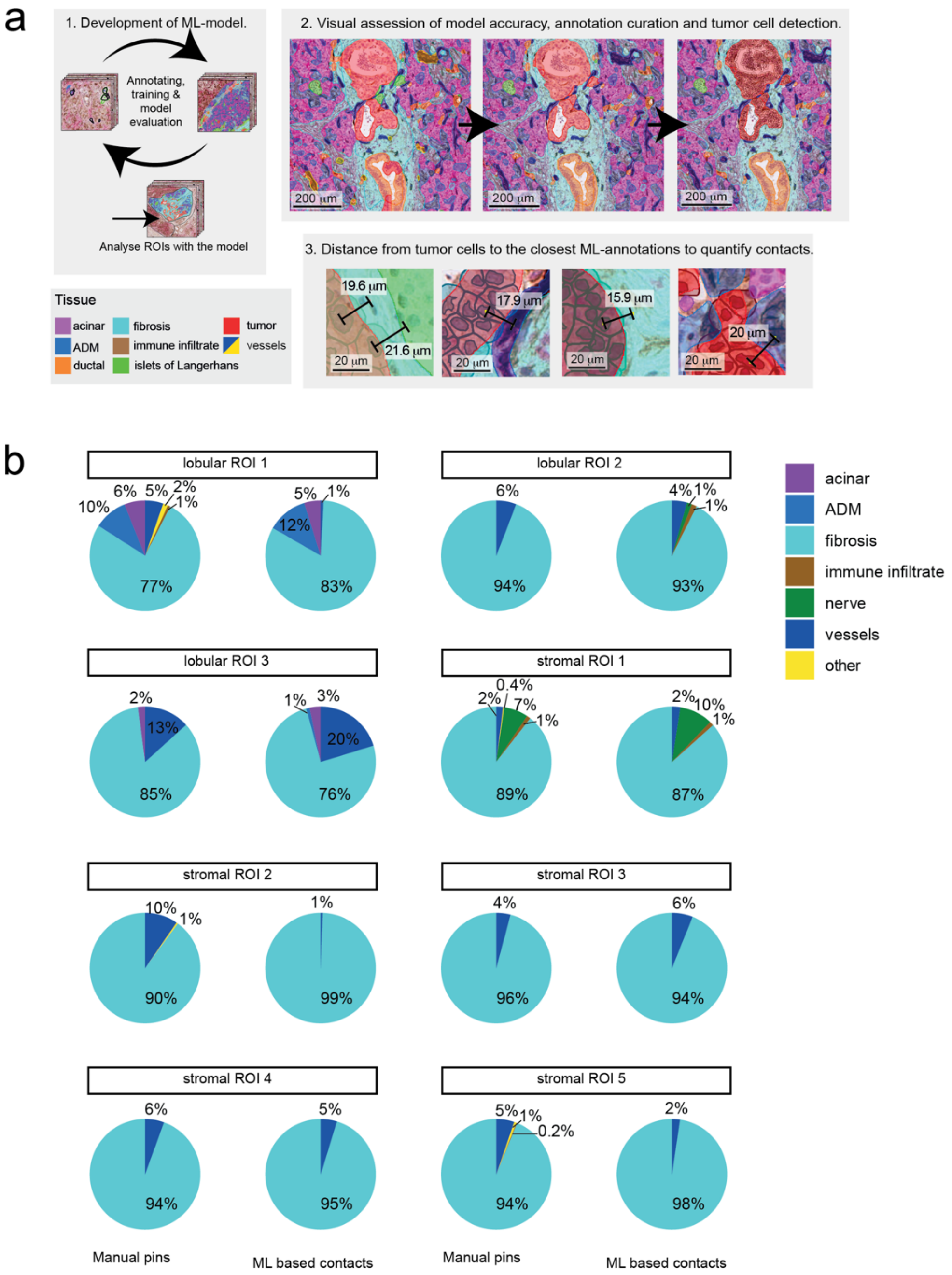

Supplementary Figure 11: Workflow of machine learning model construction for mapping of cellular proximities. a) Schematic of model construction: (1) model development; (2) curation and analysis pipeline; (3) quantification of tumor-adjacent tissue types. Distances < 20 µm from tumor cell centroids to any nearby tissue class were considered as "tumor adjacent", as exemplified in (3). ADM: acinar-to-ductal metaplasia, ROIs: regions of interest, ML: machine-learning. **b)** Fractions of contacts detected between tumor and each non-tumor tissue class, either by manually annotating each contact (left) or determined with ML-based classifications (right). Data from *n* = 8 ROIs from *n* = 1 tumor. The class 'other' comprises manually pinned contacts to cells that were characterized by the presence of a visible nucleus but that could not be assigned a class due to challenging morphology.

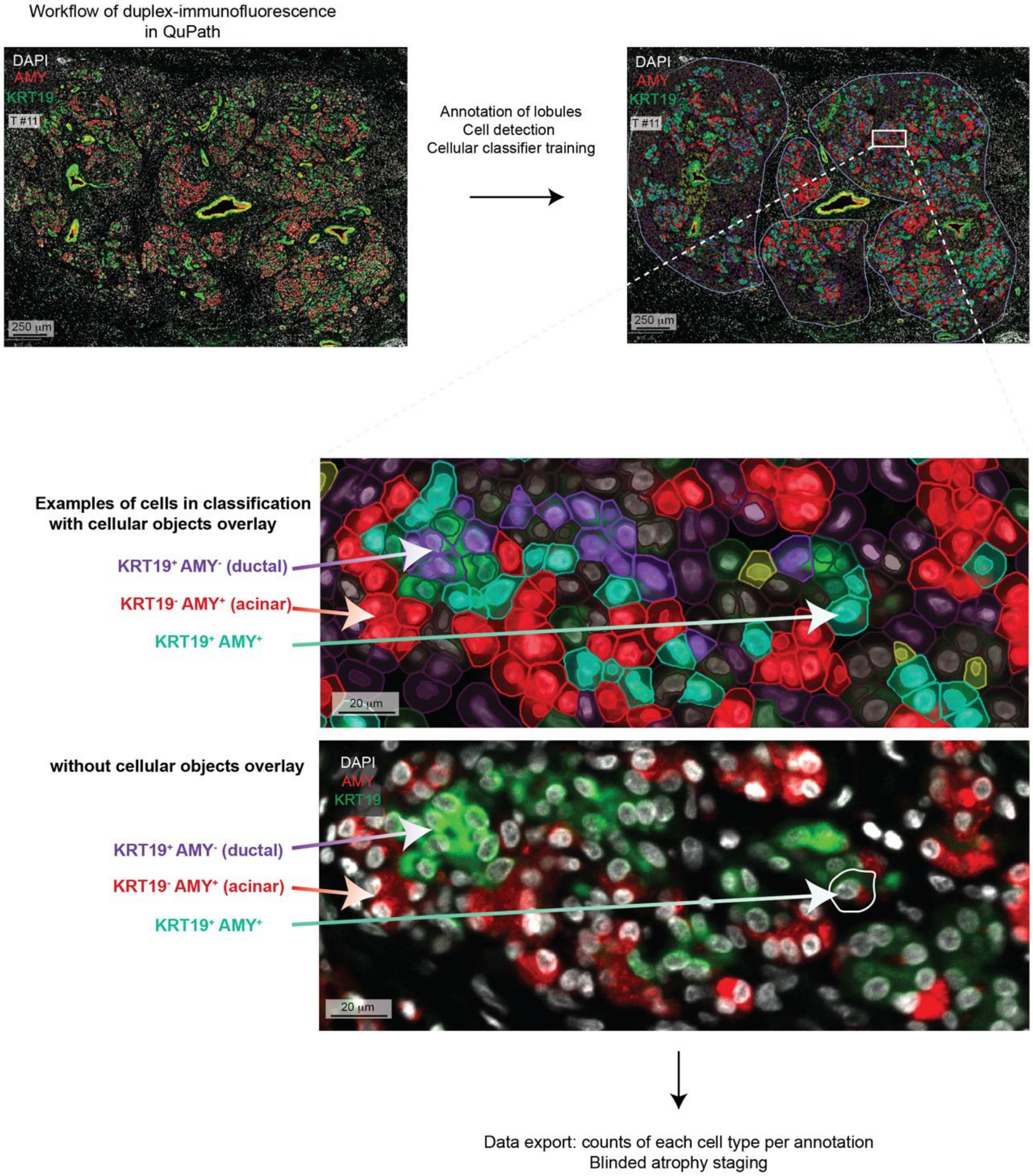

Supplementary Figure 12: Workflow of the identification of different exocrine cell types with duplex-immunofluorescence. Related to Figure 3e. Pancreatic lobules were annotated on duplex-immunofluorescence stains of α-amylase (AMY, red) and keratin (KRT)19 (green). Nuclear counterstaining was done with 4',6-diamidino-2-phenylindole (DAPI). Within lobular annotations, cell detection was performed based on the DAPI signal. Object classifiers were trained to recognize all exocrine cell types present, including the $AMY^{+}KRT19^{-}$ acinar cells, $KRT19^{+}AMY^{+}$ early acinar-to-ductal metaplasia (ADM) cells, and $KRT19^{+}AMY^{-}$ both pure ductal and late, fully transitioned ADM cells. Counts of each detected cell type were extracted from each lobule and analyzed in R.

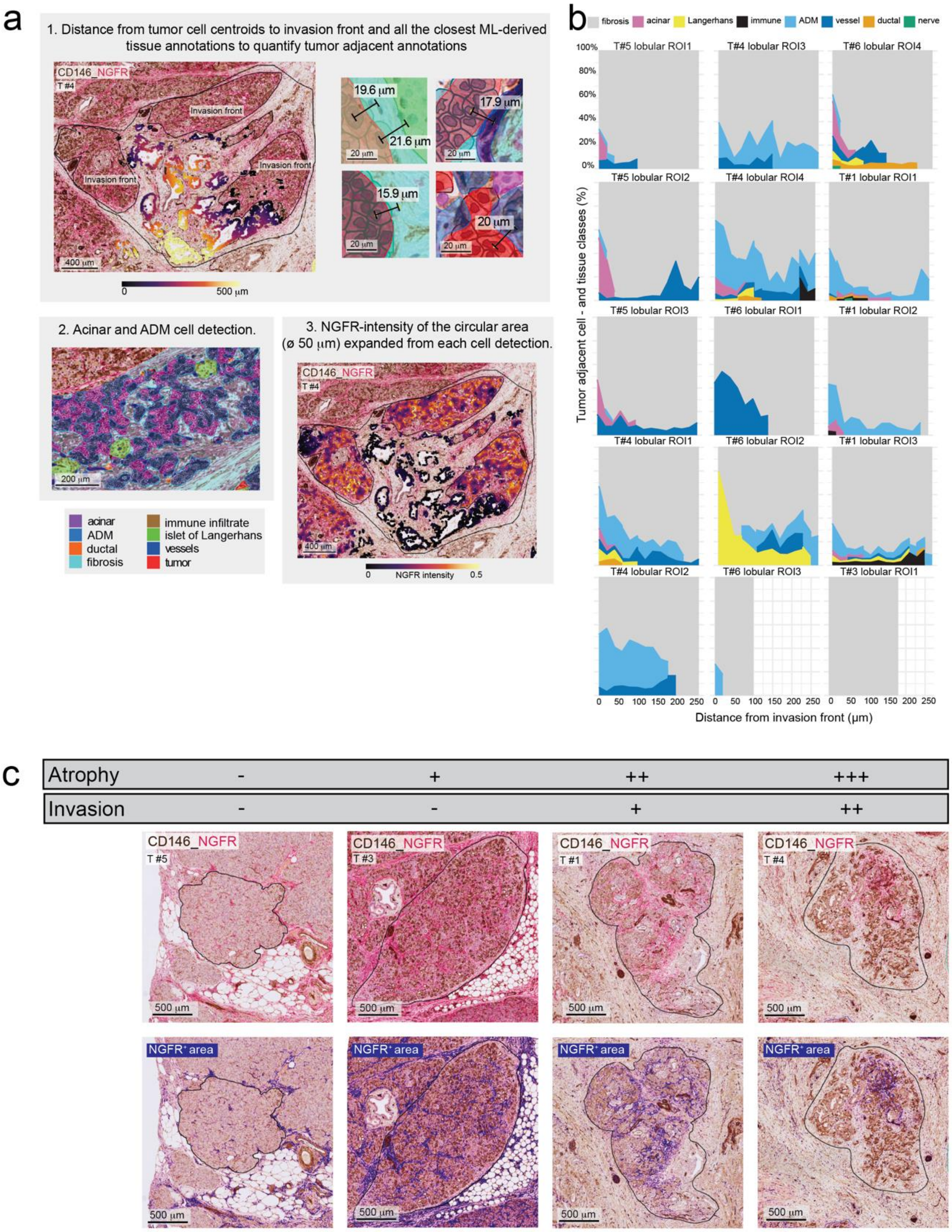

Supplementary Figure 13: Workflow of spatial interaction-based and area-based quantification of NGFR expression in the lobules. a) Based on duplex immunohistochemistry (IHC) of cluster of differentiation (CD)146 and nerve growth factor receptor (NGFR), the distances from the invasion front to tumor cells were measured, and 'contacts' between tumor cells and other classes were quantified with machine learning (ML)-based classifications. Any distance < 20 µm was considered as 'contact', as exemplified in box 1. For quantification of stromal NGFR, cells were detected inside ML-derived 'acinar' and acinar-to-ductal-metaplasia ('ADM') classifications (box 2). Thereafter, the mean NGFR stain intensity of an expanded area 50 µm in diameter from each cell detection was determined (box 3). **b)** Fractions of tumor-adjacent tissue classes within a distance of 20 µm by distance to the

lobular invasion front for all analyzed regions (*n* = 5 tumors, *n* = 15 regions) **c)** Representative images of $NGFR^+$ area-based quantification on duplex IHC of CD146 and NGFR across four lobular categories, depending on the extent of lobular atrophy and tumor invasion. Left to right: lobular categories were defined as highly intact and unaffected lobules; lobules showing signs of chronic pancreatitis but no tumor invasion; lobules with chronic pancreatitis and limited tumor invasion; and lobules with severe atrophy and extensive tumor invasion. Representative for *n* = 7 tumors. Dark blue overlay in the bottom images indicates the $NGFR^+$ area of the upper images in the panel.

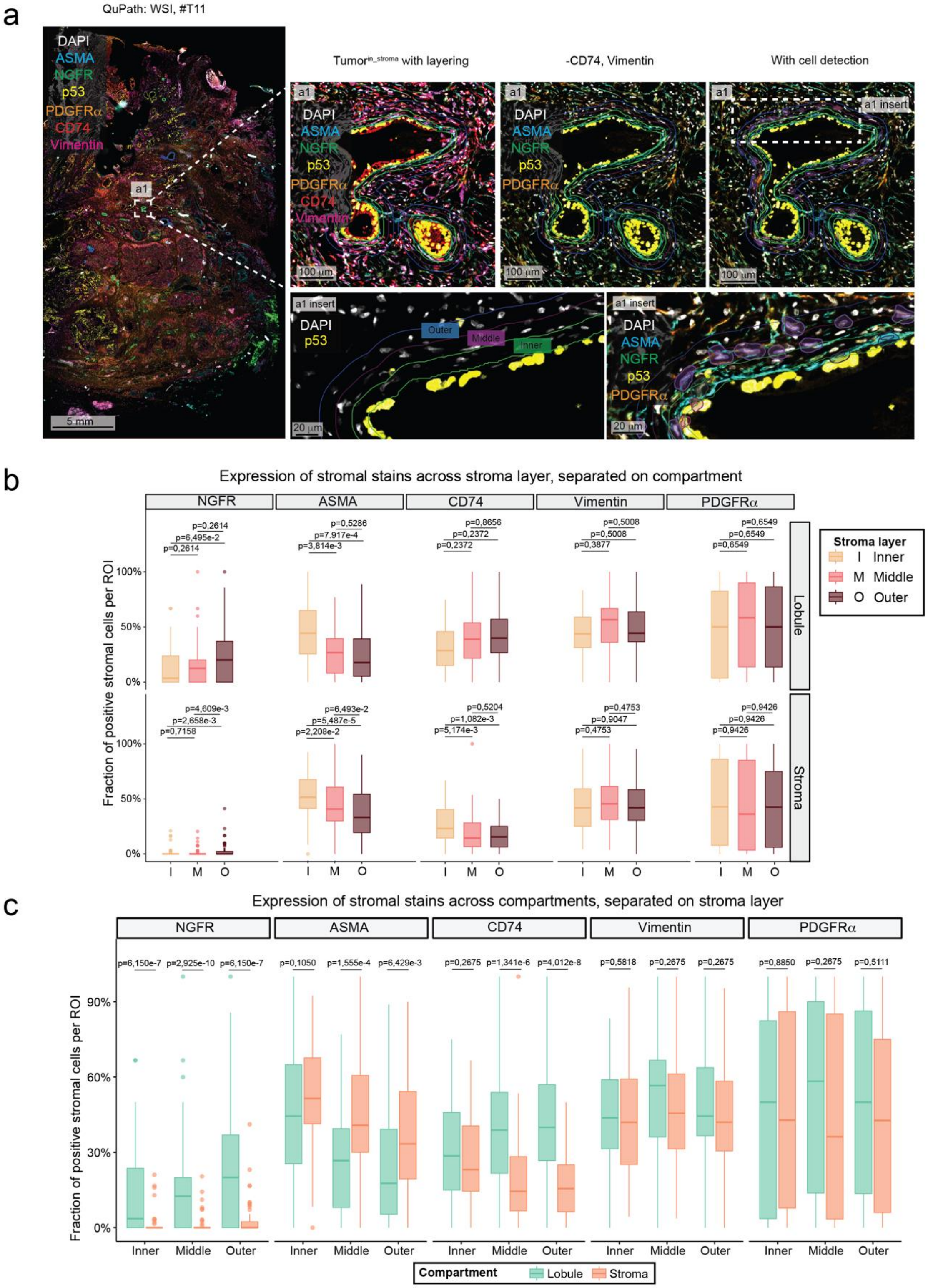

Supplementary Figure 14: Differential expression of stromal cell markers with increasing distance from tumor cells. a) Overview of the stroma layering approach. Tumor protein (p53)$^{+}$ tumor nests (yellow, nuclear) were annotated, from where three stromal layers were created by 15 µm-thick consecutive expansions: inner (closest to the tumor), middle, and outer (furthest away from the tumor). **b)** Fractions of stromal cells expressing nerve growth factor receptor (NGFR), alpha-smooth muscle actin (ASMA), cluster of differentiation (CD)74, vimentin, and

platelet-derived growth factor receptor alpha (PDGFRα) at the three distance layers from tumor nests, grouped by $tumor^{in\_lobules}$ and $tumor^{in\_stroma}$. Two-sided unpaired Kruskal-Wallis rank sum test, with post-hoc Dunn's test for pairwise multiple comparisons with Benjamini-Hochberg (BH) correction for multiple testing. Related to Fig. 5b. **c)** Fractions of stromal cell marker expression stratified by tissue compartment in the three stromal layers. Unpaired two-tailed Wilcoxon rank sum test, with BH correction for multiple testing. Related to Fig. 5c. **b)**, **c)**: Data from *n* = 8 tumors, with *n* = 222 stromal regions of interest (ROIs, *n* = 74 per stroma layer), and *n* = 141 lobular ROIs (*n* = 47 per stroma layer). Box-and-Whisker plots show the median (line), the interquartile range (IQR, box) and minimum and maximum values within 1.5 times IQR from the first and third quartile (whiskers). BH-corrected p-values are stated. Source data are provided as a Source Data file.

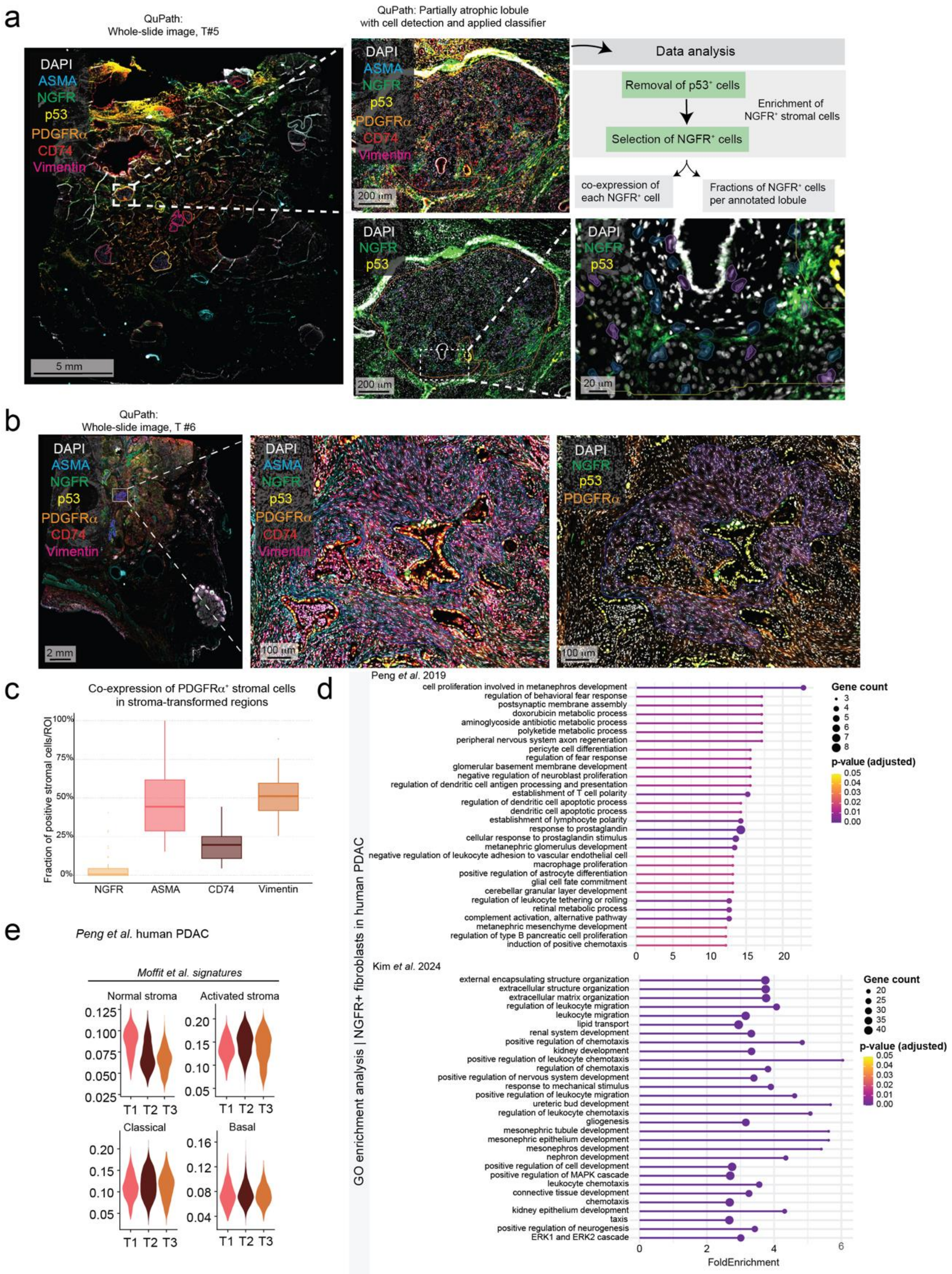

Supplementary Figure 15: Workflow for determining expression profiles of stromal cells in lobular and desmoplastic regions and co-expression analysis of PDGFRα⁺ stromal cells. a) Representative image and schematic of the workflow to analyze nerve growth factor receptor (NGFR)⁺ stromal cells present in pancreatic lobules. Whole-lobule annotations (thin lines), in which cells were detected, were manually drawn (*n* = 53, from *n* = 8 individual tumors). Classifiers for detecting positive and negative cells for each marker were trained and applied sequentially, which rendered every detection object with a classification scheme for all markers. Cells with high

nuclear circularity and tumor protein p53 (p53) positivity were excluded to enrich for stroma cells. **b)** Representative image of analyzing platelet-derived growth factor receptor alpha (PDGFRα)$^{+}$ stromal cells present in desmoplastic regions. Stromal annotations (*n* = 40 annotations from *n* = 8 individual tumors, thin lines) were manually drawn at sizes comparable to the lobular annotations in (a), in which cells were detected. Classifiers for detecting positive and negative cells for each marker were trained and applied sequentially, which rendered every detection object with a classification scheme for all markers. Cells with high nuclear circularity and p53 positivity were excluded. **c)** Co-expression of PDGFRα^{+} stromal cells with other stroma cell markers in desmoplastic, stroma-transformed regions. Data from *n* = 8 individual tumors, comprising *n* = 40 regions of interest. Box-and-Whisker plots show the median (line), the interquartile range (IQR, box) and minimum and maximum values within 1.5 times IQR from the first and third quartile (whiskers). **d)** Gene Ontology (GO, biological processes) enrichment analysis of significantly upregulated genes in NGFR+ fibroblasts compared to other fibroblasts in human PDAC data sets (Peng *et al*.; data from *n* = 24 patients and Kim *et al.;* data from *n* = 17 patients) **e)** Moffit's stroma and tumor subtype signatures across clinical TNM scoring; T1 < 2 cm, T2 2–4 cm, T3 > 4 cm (Peng *et al*.; data from *n* = 24 patients). Source data are provided as a Source Data file.

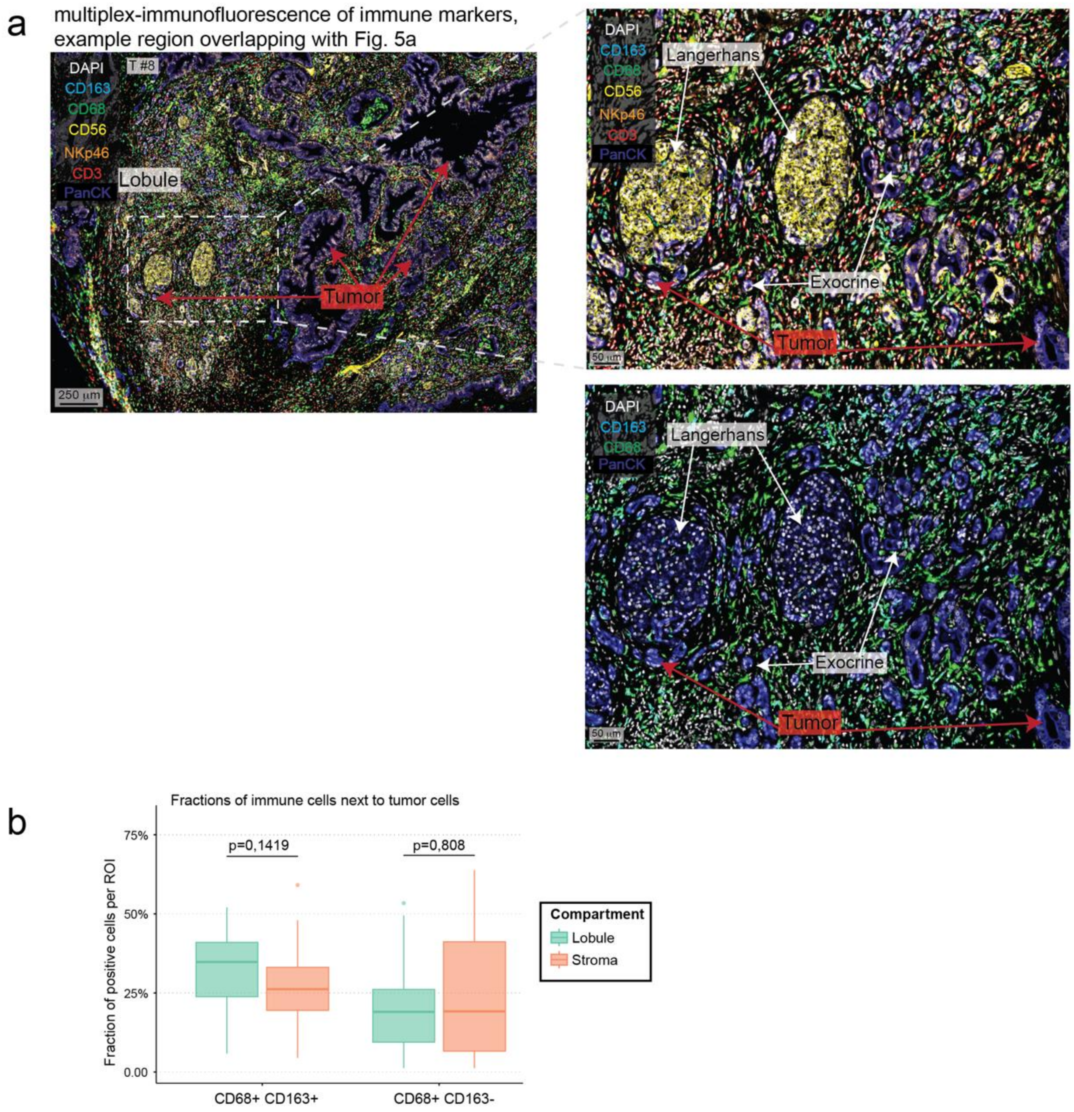

Supplementary Figure 16: Distribution of immune cells in the lobular and stromal compartments from multiplex-immunofluorescence. a) Representative multiplex-immunofluorescence of the immune markers cluster of differentiation (CD)163 (cyan), CD68 (green), CD56 (yellow), Natural killer (NK) p46 (orange), CD3 (red) and pan-cytokeratin (PanCK; blue) expressed in a lobular focal point with stroma toward right in the left panel with indicated parenchymal structures. The region is consecutive to the region in Figure 5a. Nuclear counterstaining was done with 4',6-diamidino-2-phenylindole (DAPI). **b)** Unpaired two-tailed Wilcoxon rank sum test with Benjamini-Hochberg (BH) correction for multiple testing of fractions of positive immune cells combinations next to Tumor^{in_lobule} vs. Tumor^{in_stroma}. To further enrich for macrophages, $CD3^+$ and $PanCK^+$ cells were filtered out from the analysis. *N* lobular ROIs = 34, *n* Stromal ROIs = 40. Box-and-Whisker plots show the median (line), the interquartile range (IQR, box), minimum and maximum values within 1.5 times IQR from the first and third quartile (whiskers). BH-corrected p-values are stated. Source data are provided as a Source Data file. Representative of (**a**) and data from (**b**) *n* = 8 tumors.

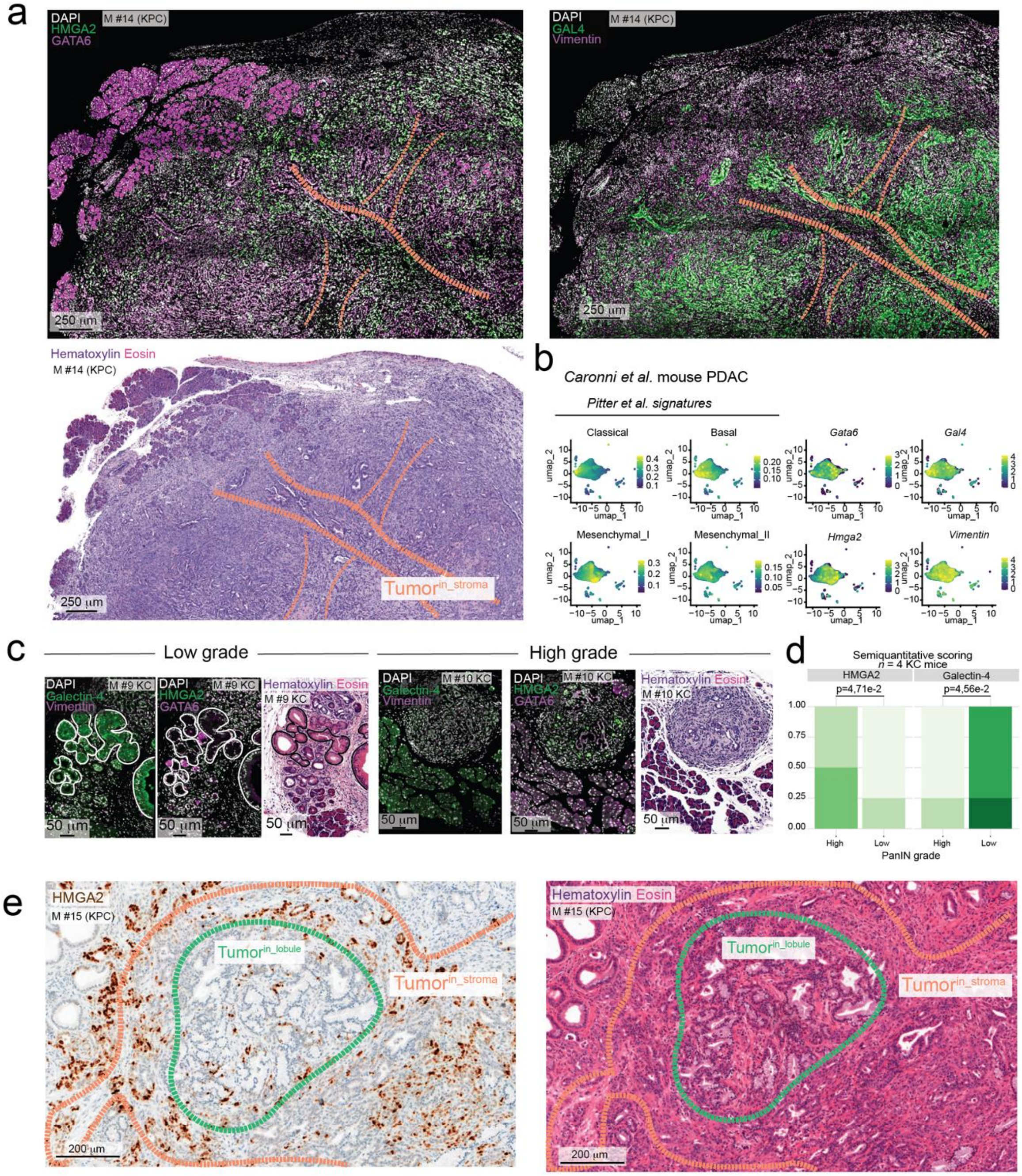

Supplementary Figure 17: Representative images of subtype marker and H&E from KPC mice. a) Representative immunofluorescence images of high mobility group AT-hook 2 (HMGA2; basal subtype marker, green left) and GATA binding protein 6 (GATA6; classical subtype marker, magenta left) and Galectin-4 (GAL4; classical subtype marker, green right) and Vimentin (basal/mesenchymal marker, magenta right), and corresponding hematoxylin and eosin (H&E, bottom). Stromal compartment, as far as discernable: dotted orange lines. **b)** Pancreatic ductal adenocarcinoma (PDAC) subtype signature expression and normalized marker gene expression in murine PDAC cells from the Caronni *et al.* dataset, related to Figure 5g&h. **c)** Representative immunofluorescence (IF) of Galectin-4 (green left), Vimentin (magenta left), HMGA2 (green middle) and GATA6 (magenta middle) and corresponding H&E stain (right) in pancreatic intraepithelial neoplasia (PanIN) in the KC mice, separated for low – and high grade PanIN. **d)** Stacked bar charts displaying the semiquantitative scorings of PanINs in KC mice for the indicated markers, using the mean score of all respective lesions for each mouse. Unpaired two-tailed Wilcoxon rank sum test with Benjamini-Hochberg (BH) correction for multiple testing. BH-corrected p-values are stated.

Related to Figure 6. **e)** Murine PDAC: Expression of HMGA2 (Immunohistochemistry, left panel) by tumor cells located in the stromal compartment (orange dotted line) and lobular compartment (green dotted line), and a consecutive H&E section (right panel). **a), c), e):** Representative of and **d)** data from *n* = 4 tumors. **b)** Data from *n* = 9 mice, Caronni *et al*. Source data are provided as a Source Data file.

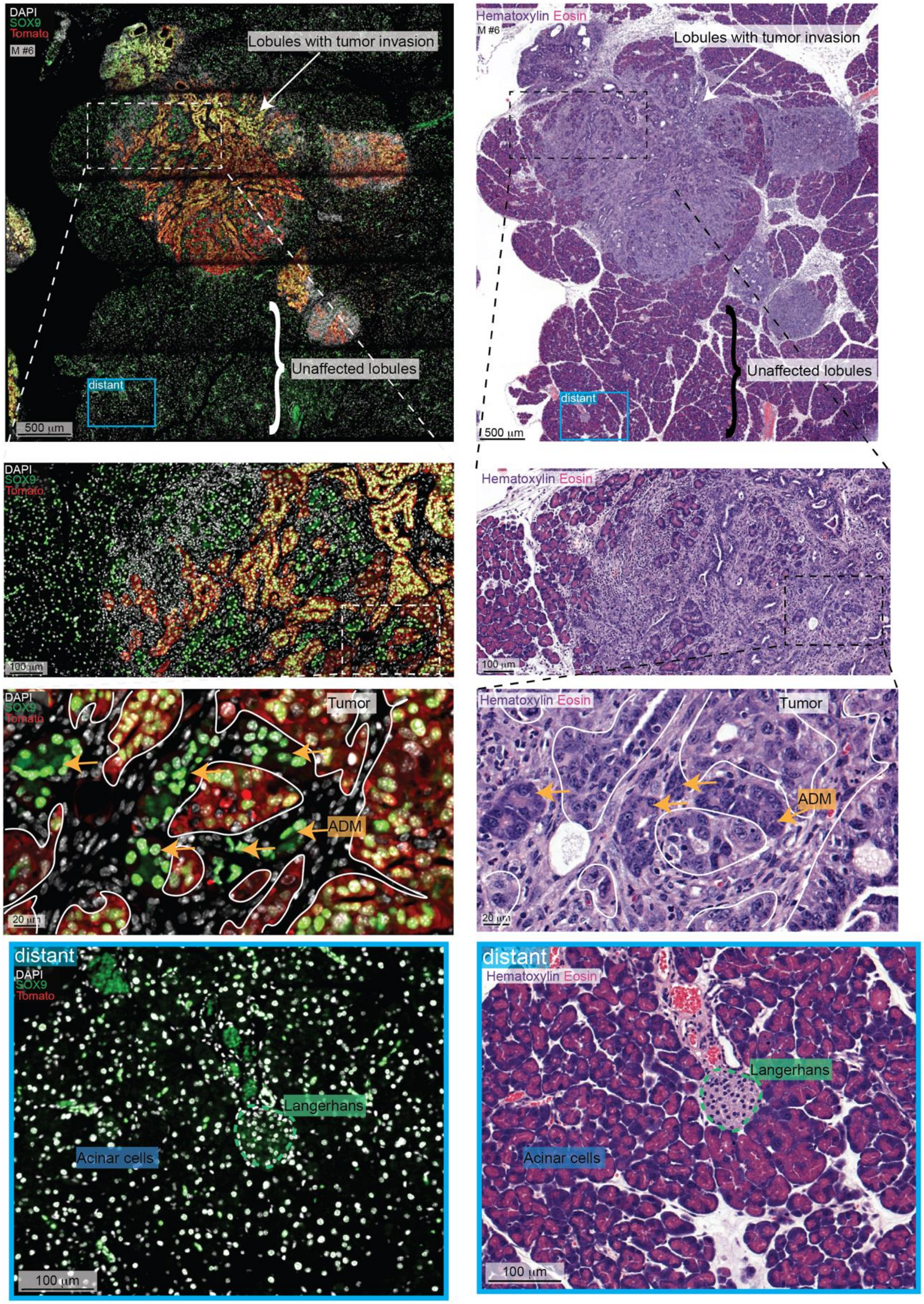

Supplementary Figure 18: Immunofluorescence of SOX9 and tdTomato in the murine orthotopic injection model. Representative immunofluorescence images of SRY-Box transcription factor 9 (SOX9; green, nuclear) and Red Fluorescent Protein/Tomato (red, cytoplasmic) in a region with lobular invasion (left panels), and hematoxylin

and eosin stain on a consecutive section (right panel). In low magnification (upper panels), the lobular tumor-infiltrating area can be seen in the upper parts and close to normal lobules, more distant from the invasion front at the lower edges. In higher magnification (middle panels), $tdTomato^+$ tumor cells are intermingling with the $Tomato^-$ lobular remnants; $SOX9^+$ cells in the process of acinar-to-ductal metaplasia (ADM; orange arrows). Lobules more distant from the active tumor invasion (lower panel; delineated with cyan) are dominated by untransformed, $SOX9^-$ acinar cells. Green dotted line: endocrine islet of Langerhans. Nuclear counterstaining was done with 4’,6-diamidino-2-phenylindole (DAPI). Representative of *n* = 2 tumors. Related to Figure 6.

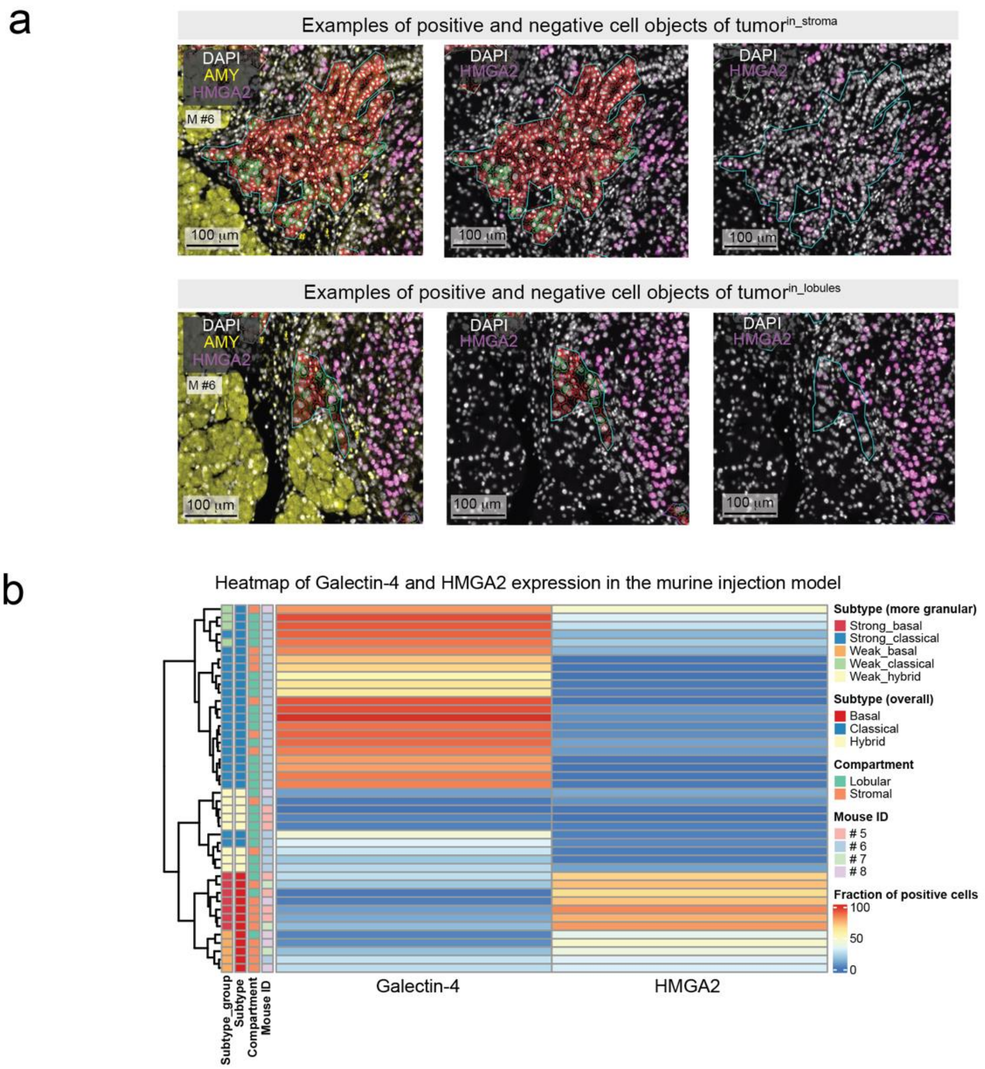

Supplementary Figure 19: Tumor subtype markers in murine tumors from the orthotopic injection model. a) Representative immunofluorescence images of high mobility group A2 (HMGA2; pink, nuclear) and α-amylase (AMY, yellow, cytoplasmic) of a mouse tumor from the orthotopic injection model showing stromal invasion (upper panel) and lobular invasion (lower panel). Thin lines delineate quantified regions of interest, red detection objects: $HMGA2^-$ tumor cells, green detection objects: $HMGA2^+$ tumor cells. **b)** Heatmap and unsupervised clustering of the indicated markers, related to Figure 6. Each heatmap cell represents the fraction of positive tumor cells for a given mouse and marker for a given region of interest (ROI). ROIs were assigned subtype based on the expression of Galectin-4 and HMGA2. Metadata indicate 1) the overall subtype assigned to the ROI (basal, hybrid, or classical) 2) a more granular subtype assignment depending on low or high expression of Galectin-4 and HMGA2 (strong and weak for all overall subtypes. No ROIs could be identified as 'Weak_hybrid') 3) Compartment identity and 4) the mouse ID. Representative of (**a**) and data from (**b**) *n* = 4 tumors.

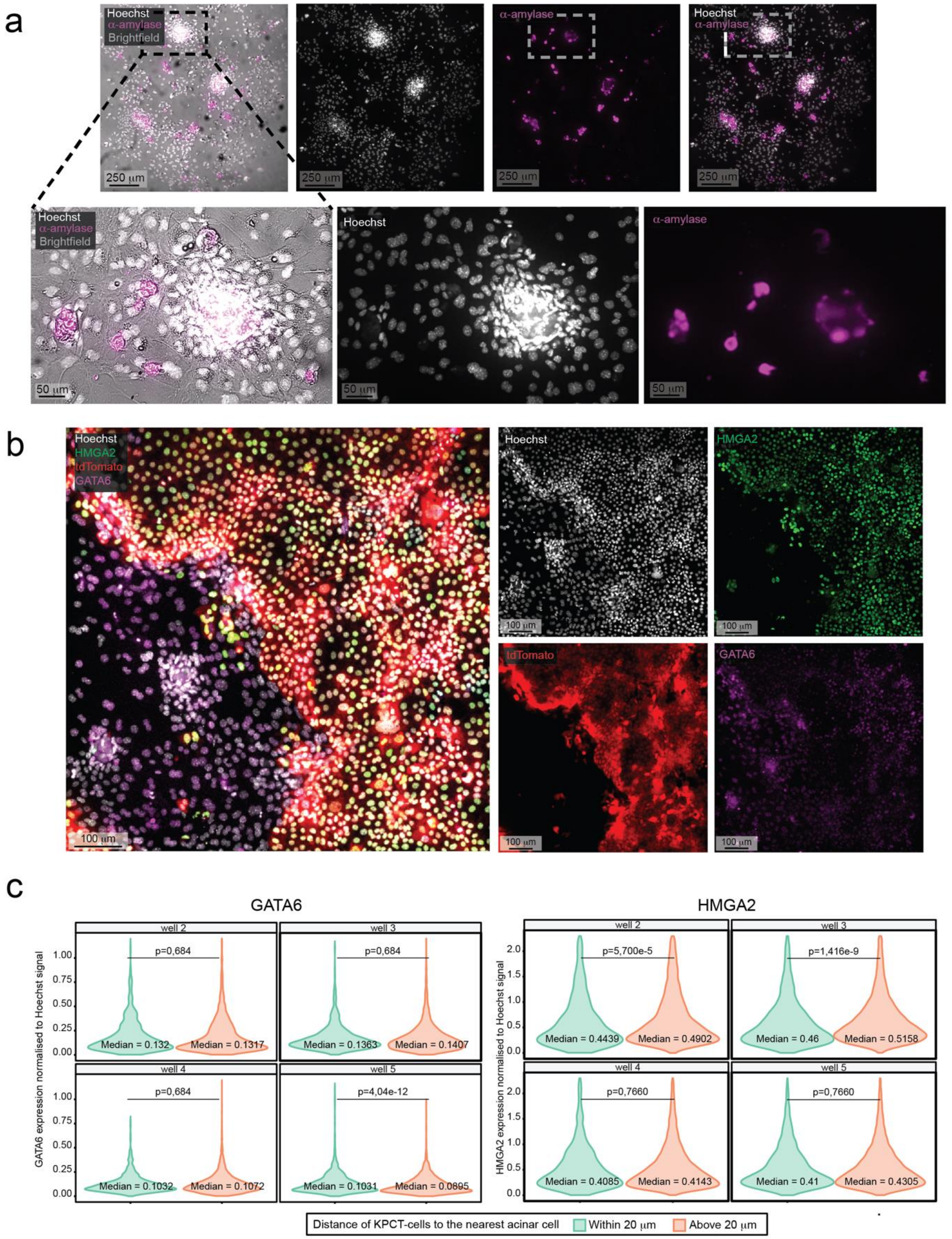

Supplementary Figure 20: Examples of the acinar-KPCT cocultures and violin plots of all wells. a) Representative immunofluorescence (IF) of α-amylase$^+$ (magenta) acinar cells after six days in culture. **b)** Representative IF of cocultures where GATA binding protein 6$^+$ (GATA6, magenta) acinar cells are seen toward the lower left, with an interface to the tdTomato+ (red) GATA6$^+$ KPCT cells, co-stained with high mobility group A2 (HMGA2, basal subtype marker, green) expression of the KPCT cells. **a), b)** Nuclear counterstaining was done with

Hoechst, which is displayed in white. **c)** Violin plots of nuclear intensity of respective markers for each tumor cell in *n* = 4 individual wells from acinar and KPCT cocultures. The defined groups are KPCT cells in the immediate vicinity to the acinar cell border ('Within 20 µm', green. Well 2: *n* = 1215 cells. Well 3: *n* = 2033 cells. Well 4: *n* = 1181 cells. Well 5: *n* = 1844 cells) and KPCT cells further from the acinar cells ('Above 20 µm', orange. Well 2: *n* = 6002 cells. Well 3: *n* = 9566 cells. Well 4: *n* = 7525 cells. Well 5: *n* = 6884 cells.). The marker intensity was normalized to the nuclear counterstain, Hoechst. Unpaired two-tailed Wilcoxon rank sum test with Benjamini-Hochberg (BH) correction. BH-corrected p-values are given. Source data are provided as a Source Data file. Related to Figure 6k.

Supplementary Table 1. Antibodies and pretreatment conditions for immunohistochemistry. The pretreatment for all antibodies was Bond Epitope retrieval solution 2 EDTA, which was applied for 20 minutes. NCL: Novocastra Leica Biosystems Ltd, Newcastle Upon Tyne, United Kingdom. MUC5AC: mucin 5AC oligomeric mucus/gel forming. CDX2: caudal type homeobox 2. KRT: Keratin. HMGA2: high mobility group AT-hook 2. CA: Carbohydrate antigen. NGFR: nerve growth factor receptor.

**Antibody**	**Clone**	**Manufacturer-product code**	**Dilution**
MUC5AC	CLH2	1:NCL-MUC-5Ac	1:50
CDX2	AMT28	NCL-CDX2	1:25
KRT17	E3	NCL-CK17	1:25
KRT5	XM26	NCL-L-CK5	1:100
CA125	CA125	NCL-L-Ca125	1:100
HMGA2	D1A7	Cell Signaling 8179	1:100
NGFR	Polyclonal	Sigma-HPA004765	1:500

Supplementary Table 2. Number of ROIs that could be included for each case in the spatially dependent quantification and notes of changes across stains. *Indicated regions of interest (ROIs) were selected by square annotations, within which manual selections were performed. The rest were selected by individual dot annotations.

**Tumor ID**	**Number of tumor^{in_lobule} ROIs**	**Number of tumor^{in_stroma} ROIs**	**Comment of ROI setting**
**T #4**	9	9	all annotations *.
**T #5**	9	9	5 lobules selected with dots, all other *
**T #16**	9	9	all annotations *.
**T #20**	9	9	
**T #19**	4	4	
**T #8**	9	9	
**T #17**	9	9	
**T #12**	5	9	All annotations *.
**T #13**	9	9	
**T #14**	9	9	All annotations *.
**T #15**	7	9	
**T #11**	9	9	
**T #6**	6	9	
**T #18**	9	9	
**T #2**	9	9	
**T #23**	9	9	
**T #24**	7	9	
**T #3**	6	9	
**T #25**	3	9	
**T #26**	9	9	
**T #21**	7	9	
**T #22**	2	4	
**T #27**	6	9	
**T #28**	9	9	
**T #30**	9	9	
**T #7**	5	9	
**T #32**	4	4	
**T #33**	9	9	
**T #9**	8	9	
**T #29**	4	9	
**T #31**	6	9	

Supplementary Table 3. Convolutional neural network generated with Aiforia Create v 5.5: model details, hyperparameters and error metrics. ADM: acinar-to-ductal metaplasia.

Model objective		Recognition of main structures of pancreas
Ground truth		Acinar cells, acinar cells undergoing ADM, ducts, stroma, large vessels, small vessels, nerves, immune cells, islets of Langerhans, tumor cells
Type (semantic segmentation)		Region
Complexity		Extra complex
Field of view		80 µm
Training regions		646
Iterations		2395 out of 3000
Training parameters	Weight decay	0.0001
	Mini batch size	0
	Mini batches per iteration	20
	Iterations without progress	100
	Initial learning rate	1
Image augmentation	Scale (min/max)	-1/1
	Aspect ratio	1
	Maximum shear	1
	Luminance (min/max)	-1/1
	Contrast (min/max)	-1/1.01
	Max with balance change	1
	Noise	0
Total area error % (FP/FN)		3.46 (0.15/0.20)
Precision %		98.37
Sensitivity %		97.85
F1 Score %		98.11
Area error %		0.35
Accuracy %		99.65

Supplementary Table 4. Imaging details of quantified from immunofluorescence of murine PDAC in injection models, KPC and KC mice. All signals were acquired in widefield fluorescence modality with 1x1 binning at 12-bit depth. The dichromatic mirror applied for all targets was a pentaband with the wavelength ranges: 441/30; 511/26; 593/37; 684/34 and 817/66. HMGA2: high mobility group AT-hook 2. GAL4: Galectin 4. SOX9: SRY-box transcription factor 9. RFP: Red fluorescent protein.

Protein (antibody) target	Secondary antibody/fluorophore	EM wheel	Ex wheel	Laser exc (nm)
**α-amylase**	(conj.) Alexa fluor 488	511/20	Neutral Density	477
**HMGA2 (quantified)**	Alexa fluor 647	685/40	MXR00543 (CELESTA-DA/FI/TR/Cy5/Cy7-A-OFF)	638
**HMGA2 (in co-stain with GATA6)**	Alexa fluor 488	515/30	None	477
**GAL4**	Alexa fluor 488	515/30	None	477
**SOX9**	Alexa fluor 488	515/30	None	477
**tdTomato/RFP**	Alexa fluor 647	685/40	None	638
**Vimentin**	Alexa fluor 647	685/40	None	638
**GATA6**	Alexa fluor 488	685/40	None	638

Supplementary Table 5. Imaging details of targets from duplex immunofluorescence of human PDAC. All signals were acquired in widefield fluorescence modality with 1x1 binning at 12-bit depth. The dichromatic mirror applied for all targets was a pentaband with the wavelength ranges: 441/30; 511/26; 593/37; 684/34 and 817/66.

Protein (antibody) target	Keratin 19	α-amylase
**Secondary antibody/fluorophore**	Alexa fluor 488	Alexa fluor 647
**EM wheel**	511/20	685/40
**EX wheel**	Neutral Density	MXR00543 (CELESTA-DA/FI/TR/Cy5/Cy7-A-OFF)
**Laser exc (nm)**	477	638

Supplementary Table 6. Details of primary antibodies applied in the multiplex- immunofluorescence. CD: Cluster of differentiation. PDGFRα: platelet-derived growth factor receptor alpha. NGFR: nerve growth factor receptor. p53: tumor protein p53. ASMA: alpha-smooth muscle actin. NK: Natural killer.

**Antibody**	**Clone name**	**Dilution**	**Supplier name**	**Catalogue number**	**Lot number**
**CD74**	Rabbit mAb/(D5N3 I)	1:300	Cell signaling	77274T	Lot#1
**PDGFRα**	Rabbit mAb(D13C 6)	1:250	Cell signaling	5241S	Lot#4
**NGFR**	Rabbit pAb	1:500	Atlas antibody	HPA004765	Lot#000015823
**p53**	Mouse mAb(DO-7)	1:300	Leica biosystem	NCL-L-P53-D07	Lot#P53-DO7-L-CE_From 6065462
**ASMA**	Mouse mAb(1A4)	1:300	Agilent	M0851	Lot#20039209
**Vimentin**	Rabbit mAb D21H3	1:500	Cell signaling	5741T	Lot#8
**CD163**	Mouse mAb(10D6)	1:400	Novacastra	NCL-L-CD163	Lot#6071795
**CD68**	Mouse mAb(PG-M1)	1:100	DAKO	M0876	Lot#41267758
**CD56**	Mouse mAb(123C3)	1:100	DAKO	M7304	Lot#41404102
**NKp46**	Rabbit pAb(NCR1)	1:250	Invitrogen	PA579720	Lot#YL4157220 6
**CD3**	Mouse mAb(F7.2.3 8)	1:80	DAKO	M7254	Lot#41755177

Supplementary table 7. Details of analyzed data sets

GEO/GSA accession number	Analyzed samples
GSE217846	GSM6727558
	GSM6727559
	GSM6727560
	GSM672561
GSE250486	GSM7979746
	GSM7979747
	GSM7979748
	GSM7979749
GSE194247	GSM5831620
	GSM5831621
	GSM5831622
	GSM5831623
	GSM5831624
CRA001160	SAMC047072
	SAMC047073
	SAMC047074
	SAMC047075
	SAMC047076
	SAMC047077
	SAMC047078
	SAMC047079
	SAMC047080
	SAMC047081
	SAMC047082
	SAMC047083
	SAMC047084
	SAMC047085
	SAMC047086
	SAMC047087
	SAMC047088
	SAMC047089
	SAMC047090
	SAMC047091
	SAMC047092
	SAMC047093
	SAMC047094
	SAMC047095

Supplementary table 8: Imaging details of immunofluorescence of in vitro cultures. All signals were acquired in widefield fluorescence modality with 1x1 binning at 12-bit depth. The dichromatic mirror applied for all targets was a pentaband with the wavelengths ranges: 441/30; 511/26; 593/37; 684/34 and 817/66. HMGA2: high mobility group AT-hook 2. GATA6: GATA binding protein 6.

**Protein (antibody) target or fluorescent protein**	**Secondary antibody/fluorophore**	**EM wheel**	**Ex wheel**	**Laser exc (nm)**
**α-amylase** (single stain)	Alexa fluor 647	685/40	None	638
**HMGA2**	Alexa fluor 488	515/30	None	477
**GATA6**	Alexa fluor 647	685/40	None	638
**tdTomato**	-	593/40	None	546
