# Supplementary material for: An injury-associated lobular microniche is associated with the classical tumor cell phenotype in pancreatic cancer
Source: Nat Commun. 2025 Sep 26;16:8307. doi: 10.1038/s41467-025-63864-7 (PMC12475445; doi:10.1038/s41467-025-63864-7)
Supplement: Supplementary file 3 — Reporting Summary [file 41467_2025_63864_MOESM3_ESM.pdf]

nature portfolio

Corresponding author(s): Marco Gerling
Last updated by author(s): Jul 10, 2025

Reporting Summary

Nature Portfolio wishes to improve the reproducibility of the work that we publish. This form provides structure for consistency and transparency in reporting. For further information on Nature Portfolio policies, see our Editorial Policies and the Editorial Policy Checklist.

Statistics

For all statistical analyses, confirm that the following items are present in the figure legend, table legend, main text, or Methods section.

n/a	Confirmed	
☐	☒	The exact sample size (*n*) for each experimental group/condition, given as a discrete number and unit of measurement
☐	☒	A statement on whether measurements were taken from distinct samples or whether the same sample was measured repeatedly
☐	☒	The statistical test(s) used AND whether they are one- or two-sided<br>*Only common tests should be described solely by name; describe more complex techniques in the Methods section.*
☐	☒	A description of all covariates tested
☐	☒	A description of any assumptions or corrections, such as tests of normality and adjustment for multiple comparisons
☐	☒	A full description of the statistical parameters including central tendency (e.g. means) or other basic estimates (e.g. regression coefficient) AND variation (e.g. standard deviation) or associated estimates of uncertainty (e.g. confidence intervals)
☐	☒	For null hypothesis testing, the test statistic (e.g. *F*, *t*, *r*) with confidence intervals, effect sizes, degrees of freedom and *P* value noted<br>*Give P values as exact values whenever suitable.*
☒	☐	For Bayesian analysis, information on the choice of priors and Markov chain Monte Carlo settings
☒	☐	For hierarchical and complex designs, identification of the appropriate level for tests and full reporting of outcomes
☒	☐	Estimates of effect sizes (e.g. Cohen's *d*, Pearson's *r*), indicating how they were calculated

Our web collection on statistics for biologists contains articles on many of the points above.

Software and code

Policy information about availability of computer code

Data collection	The custom-made scripts used to generate the plots are available at https://github.com/gerlingm/PDAC-lobular-growth.<br>R versions 4.1.2 and 4.2.2 were used to implement the analysis and generate the plot. Aiforia Create v 5.5 and higher was used to generate the machine learning model and the machine learning based annotations. The annotations were exported with Aiforia API v 1. QuPath version 0.4.3 were used to quantify the IHC and IF stains.
Data analysis	Please see above, "Data collection". The same software and code was used for collection and analysis of data.<br><br>All R packages applied, including dependencies to run the code were: ggplot2, ggthemes, forcats, rstatix, ggpubr, RColorBrewer, wesanderson, tidyr, knitr, dplyr, reactable, data.table, reshape2, corrplot, patchwork, ComplexHeatmap, viridis, ggVennDiagram, dichromat, survminer, BioVenn, Hmisc, stringr, ggridges, hrbrthemes, circlize, pheatmap, stats, ggfortify, factoextra, tidyverse, BiocManager, renv, magrittr, scales, BiocGenerics, ggalluvial, ggforce, plyr, BiocStyle, tatami, Matrix, beachmat, DelayedArray, Rcpp, Rtatami, std, Biobase, BiocFileCache, tools, httr, rappdirs, DESeq2, airway, rtracklayer, utils, BiocIO, BiocNeigbors, List, Searcher, BiocParallel, RNAseqData.HNRNPC.bam.chr14, GenomicAlignments, org.Hs.eg.db, Rmpi, parallel, BiocSingular, biomaRt, curl, Biostrings, hgu95av2probe, hgu95av2cdf, affy, affydata, rmarkdown, bit, ff, devtools, microbenchmark, scRNAseq, scuttle, scran, scater, bluster, igraph, broom, tibble, purrr, rlang, thematic, flexdashboard, shiny, bslib, lattice, testthat, checkmate, tinytest, prettydoc, colorspace, markdown, seriation, cowplot, usethis, cpp11, pkgbuild, writable, CharacterVector, Environment, bench, base, doubles, A, remotes, covr, detail, store, cpp11test, lobstr, jsonlite, lintr, a.pkg, MyPkgDependsDataTable, datasets, DBI, RKazam, SQL, RMariaDB, glue, RSQLite, nanoarrow, arrow, keyring, dbplyr, RPostgres, odbc, bigrquery, rstudioapi, nycflights13, ADSArray, dendextend, colorspace, gplots, MASS, heatmaply, cluster, package, DendSer, NMF, dynamicTreeCut, pvclust, plotly, DOSE, dplyr, nycflights13, Lahman, dqrng, Dist, IntegerVector, sitmo, boost, random, RcppParallel, sample, extra, DT, dtplyr, edgeR, emmeans, multcomp, nlme, haven, car, mediation, sandwich, mgcv, gam, MuMIn, pbkrtest, rms, MCMCpack, insight, lme4, ordinal, bayestestR, coda, rstanarm, bayesplot, MASS, rsm, estimability, fansi, fastDummies, fgsea, org.Mm.eg.db, table, AnnotationDbi,

GEOquery, limma, msigdbr, Seurat, fitdistrplus, actuar, mc2d, GeneralizedHyperbolic, bookdown, rgenoud, knitr, fitdistrplus, foreach, iterators, randomForest, formatR, googledrive, googlesheets4, gargle, gmailr, httpuv, PKG, here, lifecycle, fs, GenomeInfoDb, Rsamtools, pasillaBamSubset, GenomicRanges, TxDb.Hsapiens.UCSC.hg38.knownGene, GenomicFeatures, GetoptLong, Getopt, sp, htmltools, ggbreak, yulab.utils, ggplotify, ggimage, aplot, gridExtra, grid, ggfun, gtable, vdiffr, vctrs, munsell, cowplot, vcd, lattice, ggimage, ggplotify, ggrastr, ggbeeswarm, sf, maps, ggrepel, ggplot2movies, ggsci, gridExtra, ggsignif, ggtangle, scatterpie, ggtree, ggtext, GlobalOptions, emo, crayon, withr, cli, profvis, readr, conflicted, rprojroot, uuid, highr, rmdformats, htmlTable, htmlwidgets, sigma, mywidget, openssl, httr2, ids, rcorpora, igraph, isoband, grDevices, KEGGREST, png, litedown, ggvis, lazyeval, leidenbase, global, TOC, xfun, lubridate, metapod, nloptr, nloptr, digest, jose, pillar, units, formattable, palmerpenguins, DiagrammeR, pkgconfig, widgetframe, listviewer, polynom, future, promises, vembedr, purr, http, shinydashboard, rstudio, pinp, Annoy, NumericMatrix, RcppML, sparklines, reactR, stringi, system, readxl, foo, reprex, reticulate, tensorflow, scipy, keras, all_py_pkgs_sans_tf, markitdown, zeallot, coro, Rhtslib, ROCR, TxDb.Hsapiens.UCSC.hg18.knownGene, RCurl, RSpectra, file, styler, rvest, repurrrsive, S4Arrays, S4Vectors, sass, colourpicker, ScaledMatrix, scattermore, scatterpie, scDblFinder, aggregation, sctransform, SingleCellExperiment, Rtsne, prng, R, terra, raster, methods, gstat, mapview, SparseArray, HDF5Array, ExperimentHub, gt, SummarizedExperiment, survival, RTCGA.clinical, survMisc, survminer, svglite, fontquiver, systemfonts, tidymodels, hms, bit64, blob, rcmdcheck, tidyselect, UCell, UCSC.utils, corpus, uwot, pizza, mapproj, prettyunits, vroom, janitor, testit, tinytex, mime, xgboost, float, drat, xml2, zlibbioc, clusterProfiler.

Each individual script file provided at our GitHub repository contains the libraries required and their version numbers.

For manuscripts utilizing custom algorithms or software that are central to the research but not yet described in published literature, software must be made available to editors and reviewers. We strongly encourage code deposition in a community repository (e.g. GitHub). See the Nature Portfolio guidelines for submitting code & software for further information.

Data

Policy information about availability of data

All manuscripts must include a data availability statement. This statement should provide the following information, where applicable:

- Accession codes, unique identifiers, or web links for publicly available datasets
- A description of any restrictions on data availability
- For clinical datasets or third party data, please ensure that the statement adheres to our policy

Source data are provided with this paper and minimal dataset in the GitHub repository. Accession codes for publicly available data sets analyzed in this study are given in the Data availability statement. Raw clinical data is not publicly available, in accordance with data protection regulations, as the data contains potentially identifying or sensitive patient information. Anonimyzed data will be made available upon request to the corresponding author to the full extent legally possible.

Research involving human participants, their data, or biological material

Policy information about studies with human participants or human data. See also policy information about sex, gender (identity/presentation), and sexual orientation and race, ethnicity and racism.

Reporting on sex and gender	Sex was determined based on the clinical charts which in most cases reflect the biological gender at birth and in individual rare cases might be self reported sex. All reports are on a group level, no individual sex is reported. Sex is given in table 1.There was no significance differences between the population characteristics and sex was not considered in the data analysis.
Reporting on race, ethnicity, or other socially relevant groupings	No data on race, ethnicity, or other social grouping were collected.
Population characteristics	This information is given in Table 1.
Recruitment	Consecutive patients operated on pancreatic adenocarcinoma were included (2017 & 2020) at Karolinska University Hospital, Stockholm, Sweden; no patients were additionally recruited. Karolinska Hospital serves all of region Stockholm and adjacent counties and patients are referred to Karolinska based on geographical location. No other clinics perform a relevant number of surgical resections for pancreatic adenocarcinoma for the investigated patient population, such that coverage of nearly all patients in this region and time frame can be assumed.
Ethics oversight	The Swedish National Ethical Review Board, Etikprövningsmyndigheten via the regional board Uppsala department 2 medicine, gave ethical approval for all the work on human samples #2020-06115 and to download, storage and analyze of publicly available pseudo-anonymized human sequencing data #2024-06892-02; informed consent was waived. The participants received no compensation.

Note that full information on the approval of the study protocol must also be provided in the manuscript.

Field-specific reporting

Please select the one below that is the best fit for your research. If you are not sure, read the appropriate sections before making your selection.

☒ Life sciences ☐ Behavioural & social sciences ☐ Ecological, evolutionary & environmental sciences

For a reference copy of the document with all sections, see nature.com/documents/nr-reporting-summary-flat.pdf

Life sciences study design

All studies must disclose on these points even when the disclosure is negative.

Sample size	No sample size calculations were performed for the human experiments, due to the limited possibilities to assume effect sizes. All consecutive patients of a predetermined time period were included. The time period (years 2017 and 2020) was randomly chosen. More detailed description of the sampling process is given in Supplementary Figure 1. Sex and/or gender was not considered in the study design.<br>For mouse experiments that assessed clonality, no power calculations were performed based on the reasoning that power calculations were not reasonable because the assumable outcome range was entirely unclear. After the unequivocal results of the initial experiment (pilot) demonstrating near-monoclonality, no further experiments were done.<br><br>The study is a single-center study, where validation from other centers or hospitals were not studied. We have included several models to test markers and hypothesis - human, patient-derived PDAC, murine PDAC and murine precursor models, and in vitro cocultures. The in vitro coculture was performed twice, and we chose to include results form the experiment with the most robust readout.
Data exclusions	No data were systematically excluded. Scoring of lobular invasion was only possible in cases with lobular structures available on the histological slides and, hence, those cases without any lobular structure (inlcuding remnants such as endocrine islets of Langerhans) were not considered representative; details are given in Supplementary Figure 1 and in the Results.
Replication	The clinical cohort was chosen to represent two different years to account for possible sampling biases. No further replication experiments were performed.
Randomization	Does not apply to human data.<br>The analyses of the human data were retrospective and inherently non-randomized.
Blinding	Researchers analyzing the IF or IHC stains were blinded to ROI selection so that other researchers determined the selection of analysis regions on Hematoxylin&Eosin staining that did not show the characteristics for quantification. Furthermore, while delineating the analysis regions on quantified images, the channel to the stain of interest was kept off to avoid bias. Details are given in the Method and Author contributions sections.

Reporting for specific materials, systems and methods

We require information from authors about some types of materials, experimental systems and methods used in many studies. Here, indicate whether each material, system or method listed is relevant to your study. If you are not sure if a list item applies to your research, read the appropriate section before selecting a response.

Materials & experimental systems

n/a	Involved in the study	
☐	☒	Antibodies
☐	☒	Eukaryotic cell lines
☒	☐	Palaeontology and archaeology
☐	☒	Animals and other organisms
☐	☒	Clinical data
☒	☐	Dual use research of concern
☒	☐	Plants

Methods

n/a	Involved in the study	
☒	☐	ChIP-seq
☒	☐	Flow cytometry
☒	☐	MRI-based neuroimaging

Antibodies

Antibodies used	Clinical antibodies are given in Supplementary Table 1. Antibodies used for multiplex immunofluorescence are listed in Methods and in Supplementary Table 7. All antibodies are given below together with their validation statements ("Validation").
Validation	Validation statement are listed below:<br>Anti-Muc-5Ac Glycoprotein Leica Biosystems #1:NCL-MUC-5Ac mouse monoclonal clone CLH2, 1:50.<br>Recommended by manufacturer for IHC on FFPE for detecting Muc-5AC glycoprotein in normal and neoplastic tissues, as an asjunct to conventional histopathology using non-immunologic histochemical stains. It has been validated on 118 normal and abnormal cases were it showed expected staining result however validation data is not provided (https://shop.leicabiosystems.com/actions/ViewProductAttachment-OpenFile?LocaleId=en_US&DirectoryPath=SDSs&FileName=pa0052.pdf&UnitName=LBS). This antibody is extensively used in clinical routine at the Department of Pathology, Karolinska University Hospital, Huddinge, Sweden. The stains that were analyzed in this study were clinical routine stains used for diagnostics. Controls, i.e. suitable varying tissues with known expression or known absence of expression, are used on-slide on some but not all sections in clinical routine.<br><br>Anti-CDX2 Leica Biosystems #NCL-CDX2 mouse monoclonal, clone AMT28, 1:25.<br>Recommended by manufacturer for immunohistochemistry detecting human CDX2 molecule. Manufacturer has validated the product on IHC were large bowel showed positive staining but validation data is not provided. This antibody is extensively used in clinical routine at the Department of Pathology, Karolinska University Hospital, Huddinge, Sweden. The stains that were analyzed in this study were clinical routine stains used for diagnostics. Controls, i.e. suitable varying tissues with known expression or known

absence of expression, are used on-slide on some but not all sections in clinical routine.

Anti-Krt17 Leica Biosystems #NCL-CK17 mouse monoclonal antibody, clone E3, 1:25.
Recommended by manufacturer for IHC on FFPE for detection of cytokeratin 17 protein in normal and neoplastic tissues as an asjunct to conventional histopathology using non-immunologic histochemical stains. It has been validated on 44 normal and abnormal tissues where it showed expected staining result, however, validation data has not been provided (https://files.leicabiosystems.com/LBS/SE/en/All?keycode=CK17-L-CE). This antibody is extensively used in clinical routine at the Department of Pathology, Karolinska University Hospital, Huddinge, Sweden. The stains that were analyzed in this study were clinical routine stains used for diagnostics. Controls, i.e. suitable varying tissues with known expression or known absence of expression, are used on-slide on some but not all sections in clinical routine.

Anti-Krt-5, Leica Biosystems#NCL-L-CK5 mouse monoclonal antibody, clone XM26, 1:100.
Recommended by manufacturer for IHC on FFPE for detection of cytokeratin 17 protein in normal and neoplastic tissues. It has been validated on 135 samples were it showed expected staining result however validation data has not been provided (https://files.leicabiosystems.com/LBS/SE/en/All?keycode=CK5-L-CE&lot-range=CK5-L-CE). This antibody is extensively used in clinical routine at the Department of Pathology, Karolinska University Hospital, Huddinge, Sweden. The stains that were analyzed in this study were clinical routine stains used for diagnostics. Controls, i.e. suitable varying tissues with known expression or known absence of expression, are used on-slide on some but not all sections in clinical routine.

Anti-CA125 Leica Biosystems #NCL-L-CA125 mouse monoclonal antibody, clone OV185:1, 1:100.
Recommended by manufacturer for IHC on FFPE for detection of ovarian cancer antigen (CA125) protein in normal and neoplastic tissues as an asjunct to conventional histopathology using non-immunologic histochemical stains. It has been validated on 136 normal and 225 abnormal tissues where it showed expected staining result, however, validation data has not been provided (https://files.leicabiosystems.com/LBS/SE/en/All?keycode=CK17-L-CE). This antibody is extensively used in clinical routine at the Department of Pathology, Karolinska University Hospital, Huddinge, Sweden. The stains that were analyzed in this study were clinical routine stains used for diagnostics. Controls, i.e. suitable varying tissues with known expression or known absence of expression, are used on-slide on some but not all sections in clinical routine.

Anti-HMGA2, Cell Signaling Technology #8179 rabbit monoclonal antibody clone D1A7, 1:100 on human, patient-derived sections and in vitro cocultures, and 1:200 on murine sections.
Recommended by manufacturer on immunohistochemistry on FFPE to recognize endogenous levels of total HMGA2 protein https://www.cellsignal.com/products/primary-antibodies/hmga2-d1a7-rabbit-mab/8179. Antibody guarantee provided by the manufacturer. Validation data for IHC shown on the manufacturer homepage include positive nuclear staining of tumor cells on human colon carcinoma.

Anti-NGFR, Sigma Aldrich #HPA004765 (provided by Atlas Antibodies applied in multiplex-immunofluorescence) rabbit polyclonal, 1:500
Recommended by manufacturer for IHC on FFPE (https://www.sigmaaldrich.com/SE/en/product/sigma/hpa004765). This antibody is extensively used in clinical routine at the Department of Pathology, Karolinska University Hospital, Huddinge, Sweden. The stains that were analyzed in this study were clinical routine stains used for diagnostics. Controls, i.e. suitable varying tissues with known expression or known absence of expression, are used on-slide on some but not all sections in clinical routine.

Anti-CD74, Cell signaling #77274T, rabbit monoclonal antibody, clone D5N3I, 1:300
Recommended by manufacturer on immunohistochemistry on FFPE to recognize endogenous levels of total CD74 protein https://www.cellsignal.com/products/primary-antibodies/cd74-d5n3i-xp-174-rabbit-mab/77274. Antibody guarantee provided by the manufacturer. Validation data for IHC shown on the manufacturer homepage include staining of cytoplasm of fibroblasts next to tumor cells on human prostate carcinoma.

Anti-PDGFRa, Cell signaling #5241S, rabbit monoclonal antibody, clone D13C6, 1:250
Recommended by manufacturer on immunohistochemistry on FFPE to recognize endogenous levels of total PDGFRa protein https://www.cellsignal.com/products/primary-antibodies/pdgf-receptor-a-d13c6-xp-rabbit-mab/5241. Antibody guarantee provided by the manufacturer. IHC validation data on the manufacturer homepage shows staining for PDGFRa on FFPE embedded PDGFRa-positive NCI-H1703 cells while no staining detected for PDGFRa-negative HCC827 cells

Anti-p53, Leica Biosystem #NCL-L-P53-D07, mouse monoclonal antibody, clone DO-7, 1:300
Recommended by manufacturer for IHC on FFPE for detection of human p53 protein in normal and neoplastic tissuesas, an adjunct to conventional histopathology using non-immunologic histochemical stains. It has been validated on 44 samples on normal and 463 samples of malignant tisuue were it showed expected staining result, however, validation data has not been provided. (https://files.leicabiosystems.com/LBS/SE/en/All?keycode=P53-DO7-L-CE&lot-range=P53-DO7-L-CE)

Anti-ASMA, Agilent #M0851, mouse monoclonal antibody, clone 1A4, 1:300
Recommended by manufacturer for IHC on FFPE; antibody guarantee provided by the manufacturer; validation data for IHC shown on the manufacturer homepage include positive cytoplasmatic staining of stromal cells and negative epithel on human colon; antibody homepage: https://www.agilent.com/en/product/immunohistochemistry/antibodies-controls/primary-antibodies/actin-%28smooth-muscle%29-%28concentrate%29-76542
This antibody is extensively used in clinical routine at the Department of Pathology, Karolinska University Hospital, Huddinge, Sweden. The stains that were analyzed in this study were clinical routine stains used for diagnostics. Controls, i.e. suitable varying tissues with known expression or known absence of expression, are used on-slide on some but not all sections in clinical routine.

Vimentin, Cell signaling #5741T, Rabbit monoclonal antibody, clone D21H3, 1:500, for human, patient-derived sections
Recommended by manufacturer on immunohistochemistry on FFPE to recognize endogenous levels of total vimentin protein https://www.cellsignal.com/products/primary-antibodies/vimentin-d21h3-xp-174-rabbit-mab/5741 Antibody guarantee provided by the

manufacturer. IHC validation data on the manufacturer homepage shows positive staining for vimentin on FFPE embedded human tonsil in presence of antigen-specific peptid while no staining detected in precense of control peptide.

Anti-Vimentin, Santa Cruz Biotechnology, #sc7557, affinity purified goat polyclonal antibody, clone C-20, 1:50, for murine sections
Recommended by manufacturer for immunofluorescence (IF) on FFPE to recognize endogenous levels of the intermediate filament Vimentin protein in the dilution range 1:50-1:1000. https://datasheets.scbt.com/sc-7557.pdf. Validation data for IF shown on the manufacturer homepage include cytoplasmic staining of methanol-fixed A10 cells, immunoperoxidase staining of FFPE human breast tumor with cytoplasmic staining.

anti-GATA6, R&D Systems, #AF1700, goat polyclonal antibody, 1:80
Recommended by manufacturer for immunocytochemistry and immunohistochemistry to recognize human GATA6 protein in the concentration range 5-15 µg/mL (https://resources.rndsystems.com/pdfs/datasheets/af1700.pdf?v=20250430). Validation data for IF shown on the manufacturer homepage include nuclear staining of blastocysts, and nuclear staining of human GATA6 on FFPE sections at 5 µg/mL.

anti-SOX9, Sigma, #AB5535, rabbit polyclonal antibody, 1:100
Recommended by manufacturer for immunocytochemistry and immunohistochemistry to recognize human SOX9 protein (https://www.sigmaaldrich.com/SE/en/product/mm/ab5535#product-documentation). Validation data for IF shown on the manufacturer homepage include quality evaluation by Western blotting in Hep G2 cell lysate in 1:2000 dilution, however images are not directly provided. The antibody has been continuously tested in research application.

anti-Red fluorescent protein (RFP)/tdTomato, Nordic Biosite, #ASJ-JJLIOE-150, goat polyclonal antibody, 1:100
Recommended by manufacturer for immunofluorescence and immunohistochemistry to recognize tdTomato/RFP protein in the concentration range 1:50-1:500 (https://nordicbiosite.com/product/ASJ-JJLIOE-150/RFP). Validation data for IF provided by the manufacturer state reactivity with tdTomato on brain sections in IHC, and cds plasmid-transfected HEK293 cells which results in a band at 55kDa, however images are not directly provided. The antibody has been continuously tested in research application.

Anti-Galectin-4, Invitrogen, #PA5-34913, rabbit polyclonal antibody, 1:400.
Recommended by manufacturer for immunohistochemistry of FFPE material to recognize human, mouse and rat Galectin-4 protein in the concentration range 1:100-1:1000 (https://www.thermofisher.com/order/genome-database/dataSheetPdf?producttype=antibody&productsubtype=antibody_primary&productId=PA5-34913&version=Local). Validation data for IF provided by the manufacturer demonstrate reactivity with Galectin-4 on human gastric cancer of paraffin-embedded sections in IHC at 1:500 dilution with EDTA-based antigen retrieval buffer.

Anti-CD163 Leica Biosystems NCL-L-CD163 mouse monoclonal antibody (clone 10D6), 1:400.
Recommended by manufacturer for IHC on FFPE for detection of CD163 protein in normal and neoplastic human tissues. It has been validated on 43 normal and 211 abnormal tissues where it showed expected staining result. Validation data has not beenprovided https://files.leicabiosystems.com/LBS/SE/All/en?keycode=CD163-L-CE&lot-range=CD163-L-CE.

Anti-CD68, DAKO, M0876, mouse monoclonal antibody (clone PG-M1), 1:100.
The antibody labels COS-1 and WOP cells transfected with CD68 cDNA. Unlike other CD68 antibodies, which label both macrophages and myeloid cells, the PG-M1 antibody detects a fixative-resistant epitope on the macrophage-restricted form of the CD68 antigen. Recommended by manufacturer for IHC on FFPE for detection of CD68 protein in normal and neoplastic human tissues. It has been validated on wide range of normal and over 400 abnormal tissues where it showed expected staining result but validation data has not beenprovided. https://www.agilent.com/en/library/eifu?partNumber=M087601-2

Anti-CD56, DAKO, M7304, mouse monoclonal antibody (clone 123C3), 1:100.
Cells labeled by the antibody display cytoplasmic and/or membrane staining. Recommended by manufacturer for IHC on FFPE, FF and smear for detection of CD68 protein in normal and neoplastic human tissues. It has been validated on wide range of normal and over 100 abnormal tissues where it showed expected staining result but validation data has not been provided. Intended for in vitro diagnostic use. https://www.agilent.com/en/library/eifu?partNumber=M087601-2

Anti-NKp56, #PA5-79720, Invitrogen, rabbit polyclonal antibody, 1:250
NKp46 (CD335, NCR1,) is a cytotoxicity-activating receptor that may contribute to the increased efficiency of activated natural killer (NK) cells to mediate tumor cell lysis. NKp46 consists of two Ig-like domains assembled to leukocyte immunoglobulin-like (LIR) and killer inhibitory receptors (KIR). Product tested by manufacturer for IHC on FFPE on detection of NKp56 protein in human, mouse and rat normal and neoplastic tissues. Product is cited in many publications and example images of expected staining result is provided by the manufacturer https://www.thermofisher.com/antibody/product/NKp46-CD335-Antibody-Polyclonal/PA5-79720.

Anti-CD3, DAKO, mouse monoclonal antibody (clone F7.2.38), 1:80.
Cells labeled by the antibody display membranous and/or cytoplasmic staining. Recommended by manufacturer for IHC on FFPE, FF and cell preparations for detection of CD3 protein in normal and neoplastic human tissues. It has been validated on wide range of normal and over 50 abnormal tissues where it showed expected staining result but validation data has not been provided. Intended for in vitro diagnostic use https://www.agilent.com/en/library/eifu?partNumber=M087601-2.

Anti-alpha-amylase #A8273, Sigma-Aldrich, rabbit polyclonal antibody, 1:100.
The antiserum is specific for human a-amylase found in human saliva and human pancreatic extract. According to manufactorer, no reaction with other human saliva proteins or pancreatic extract proteins is observed. Product has been used in IHC and IF analyses of FFPE samples from mice and human https://www.sigmaaldrich.com/SE/en/product/sigma/a8273?srsltid=AfmBOooH4m_nawgfumB4aSAOtBXqkF5P4sYE2llXE5Q4A3M_xSb-zNZP.

Anti-KRT19, #61010, Progen, mouse monoclonal antibody, (clone Ks19.1 (A53-B/A2)), 1:20
Presents an excellent marker to discriminate glandular epithelial carcinoma from those of different origin. Tumors specifically detected: all tested adenocarcinoma; cholangio carcinoma of liver; renal cell carcinoma; transitional cell carcinoma of the bladder; ovary carcinoma; squamous cell carcinoma of cervix, bronchus and lung (intermediate type); mesothelioma; carcinoid tumor of bronchus; breast carcinoma; thymoma. The antibody has been used for human and xenopus tissues on IHC, IF for FFPE and FF samples. Product has been cited in many publications and example images of expected staining result is provided by the manufacturer https://www.progen.com/anti-Keratin-K19-mouse-monoclonal-Ks19.1-A53-B-A2-lyophilized-purified/61010.

Eukaryotic cell lines

Policy information about cell lines and Sex and Gender in Research

Cell line source(s)	KPCT cells were directly derived from KPCT mice in-house in earlier studies. The strains to generate KPC-T cells were: KrasLSL-G12D/+;Trp53LSL-R172H/+;Pdx-Cre (Hingorani, Cell, 2005, PMID 15894267); B6.Cg-Gt(ROSA)26Sortm9(CAG-tdTomato)Hze/J.<br><br>Primary pancreatic acinar cells isolated from two female C57BL/6J mice, whereof acinar cells from one mouse were applied to generate the manuscript figure.
Authentication	KPC-T: proliferation, morphology, expression of tdTomato and Keratin 19 as seen by immunofluorescence.<br><br>Primary acinar cells: morphology, clustering formation and expression of alpha-amylase seen by immunofluorescence.
Mycoplasma contamination	Mycoplasma is routinely controled for in the cell culture lab, but not in these cultures specifically.
Commonly misidentified lines (See ICLAC register)	*Name any commonly misidentified cell lines used in the study and provide a rationale for their use.*

Animals and other research organisms

Policy information about studies involving animals; ARRIVE guidelines recommended for reporting animal research, and Sex and Gender in Research

Laboratory animals	Injection model: C57BL/6J mice obtained from Charles River were used for all injection experiments. Female mice at 9-11 weeks of age were included. Mice were housed in specific-pathogen-free conditions at a 12h light (dark cycle at approximately 20-22°C). Mice had ad libitum access to standard chow (Mucedola, #2918) and water.<br><br>KPC mice: KrasLSL-G12D/+;Trp53LSL-R172H/+;Pdx-Cre. One female and three male, euthanised at 2-5 months of age.<br><br>KC mice: KrasLSL-G12D/+;Pdx-Cre on a C57BL/6 background. Four female, euthanised at 6 months of age.
Wild animals	No wild animals were used.
Reporting on sex	All mice in the injection model and KC model were female as reported in Methods. This was based on the availability of mice and no additional efforts were made to include male mice. One female KPC mouse and three male KPC mice were included for the same reasons. No sex-based analyses were applied due to the limited sample size.
Field-collected samples	No field-collected samples were used.
Ethics oversight	The Swedish Board of Agriculture approved the animal experiments via the regional ethics committees, "Linköpings djurförsöksetiska nämd" (#00217-2022 for the injection models) and "Stockholms Södra djurförsöksetiska nämnd" #S66-14 for the KC mice. Authochtonous KPC mice were analyzed and have been reported previously, Strell, C., Norberg, K., Mezheyeuski, A. et al. Stroma-regulated HMGA2 is an independent prognostic marker in PDAC and AAC. Br J Cancer 117, 65–77 (2017). https://doi.org/10.1038/bjc.2017.14 and was covered by permit #S31-15 (to Rainer Heuchel, issued by "Stockholms Södra djurförsöksetiska nämnd", 2015.

Note that full information on the approval of the study protocol must also be provided in the manuscript.

Clinical data

Policy information about clinical studies
All manuscripts should comply with the ICMJE guidelines for publication of clinical research and a completed CONSORT checklist must be included with all submissions.

Clinical trial registration	A clinical trial registration does not apply, since this is a retrospective cohort study where clinical data were used for sample identification. Not RCT, hence CONSORT does not apply.

Study protocol	This does not apply, retrospective cohort study. All details are entirely given in the Methods section. No preregistration was performed due to the simple retrospective study design.
Data collection	Patients with pancreatic adenocarcinoma operated 2017 and 2020 were included. All consecutive patients 18 years of age or older, operated at Karolinska University Hospital Huddinge were included. All patients had histopathologically confirmed PDAC.
Outcomes	Outcome measured are not reported.

Plants

Seed stocks	does not apply
Novel plant genotypes	does not apply
Authentication	does not apply
