# Supplementary material for: An injury-associated lobular microniche is associated with the classical tumor cell phenotype in pancreatic cancer
Source: Nat Commun. 2025 Sep 26;16:8307. doi: 10.1038/s41467-025-63864-7 (PMC12475445; doi:10.1038/s41467-025-63864-7)
Supplement: Supplementary file 4 — Description of Additional Supplementary Files [file 41467_2025_63864_MOESM4_ESM.pdf]

Description of Additional Supplementary Files

File name: Supplementary Data 1

Description: Script commands and image settings applied to the image analysis performed in QuPath.
